# Metal-sensing properties of the disordered loop from the Arabidopsis metal transceptor IRT1

**DOI:** 10.1042/BCJ20240685

**Published:** 2025-05-06

**Authors:** Virginia Cointry, Reyes Ródenas, Nelly Morellet, Steven Fanara, Valérie Cotelle, Julie Neveu, Grégory Vert

**Affiliations:** 1 Plant Science Research Laboratory (LRSV), UMR5546 CNRS/Université Toulouse 3, 24 chemin de Borde Rouge, Auzeville Tolosane 31320, France; 2 Structural Chemistry and Biology Team, Institut de Chimie des Substances Naturelles, CNRS UPR 2301, Université Paris-Sud, Université Paris-Saclay, Gif-sur-Yvette, France

**Keywords:** Arabidopsis, iron, metals, plants, sensing, transport

## Abstract

The plant iron-regulated transporter 1 (IRT1) iron transporter is a plasma membrane protein that takes up iron in the root under iron-limited conditions. Besides its primary metal substrate iron, IRT1 transports other divalent metals that overaccumulate in plants when soil iron is low and IRT1 is highly expressed. We previously reported that the intracellular regulatory loop between transmembrane helices TM4 and TM5 is involved in the post-translational regulation of IRT1 by its non-iron metal substrates. Upon excess of zinc, IRT1 undergoes phosphorylation by CIPK23 followed by its ubiquitination by IDF1 to target IRT1 for vacuolar degradation. This zinc-dependent down-regulation of IRT1 requires the presence of four histidine (H) residues in the IRT1 loop, which directly bind zinc. However, how selective metal binding is achieved and how this allows downstream regulation to take place is largely not known. Here, we characterized the metal-binding properties and structure of the IRT1 loop to better understand the molecular basis of non-iron metal sensing and signaling. Using a combination of circular dichroism and NMR, we reveal that zinc and manganese bind to the IRT1 loop with nanomolar range affinity and that metal binding does not trigger structuration of the loop. We validate that zinc and manganese binding is mediated by four H residues and identify aspartic acid (D) residue D173 as helping in metal co-ordination and participating to metal sensing and metal-dependent degradation of IRT1 in plants. Altogether, our data provide further understanding of how IRT1 regulatory loop senses high cytosolic divalent metal concentrations to regulate metal uptake in plants.

## Introduction

The first-row transition metal iron is the most abundant and used metal in cells [1]. Iron is widely used as a cofactor, participating in oxidation–reduction reactions. In plants, iron is of utmost importance due to its involvement in photosynthesis electron transfer reactions [2]. In nature, iron is often unavailable to organisms including plants because it is found as insoluble ferric oxide/hydroxide. Lack of iron deeply affects plant growth and leads to severe leaf chlorosis [2]. On the other hand, an excess of iron is detrimental to cells due to its redox properties and participation in the Fenton reaction that leads to reactive oxygen species formation [3]. For these reasons, living organisms and plants, in particular, need to tightly control metal uptake.

Dicotyledonous plants, such as the model plant *Arabidopsis thaliana*, use a three-step iron uptake process based on the acidification-based solubilization of soil ferric iron (Fe^3+^) insoluble oxides and the reduction in ferrous iron (Fe^2+^) prior to transport. This is mediated by the AHA2 proton pump, which secretes proton, and the FRO2 ferric chelate reductase, which reduces Fe^3+^ to Fe^2+^ [4–7]. In Arabidopsis, the main transporter involved in the uptake of Fe^2+^ is the iron-regulated transporter 1 (IRT1) [8], one of the founding members of the family of ZIP transporters that are found across all kingdoms of life [9]. Arabidopsis IRT1 has a broad substrate range, as it transports other essential divalent heavy metals such as zinc (Zn^2+^), manganese (Mn^2+^), cobalt (Co^2+^), and toxic cadmium (Cd^2+^) ions [8,10–16]. *In planta*, IRT1 is the major root iron transporter responsible for iron uptake from the soil under iron-limited conditions. IRT1 protein indeed localizes to the plasma membrane, and *IRT1* gene is strongly expressed in iron-deficient root epidermal cells [8,15]. Consistently, *irt1-1* knockout mutant plants are highly chlorotic and show growth impairment, a phenotype that can be reversed by the addition of high amounts of iron in watering solutions [8]. The chlorosis of *irt1* mutants is, however, not complemented by the other divalent metal substrates of IRT1, indicating that these constitute secondary substrates that are nonspecifically transported.

In recent years, the molecular mechanisms underlying the regulation of *IRT1* expression by its metal substrates have been uncovered. The transcription of *IRT1* is up-regulated upon iron starvation through a cascade of bHLH transcription factors [17–23]. Most importantly, post-translational regulation of IRT1 by its non-iron metal substrates Zn^2+^, Mn^2+^, Co^2+^, and Cd^2^^+^ has recently been reported [16,24]. Non-iron metal availability indeed regulates the subcellular localization and stability of IRT1. In the absence of non-iron metals, IRT1 sits at the cell surface to take up low available iron from the soil. Increasing non-iron metal levels lead to partial IRT1 internalization in early endosomes, as a result of its multi-monoubiquitination on lysine residues K154 and K179 by a yet-to-be-characterized E3 ubiquitin ligase [15]. Higher non-iron metal levels trigger IRT1 phosphorylation by the CIPK23 kinase and the recruitment of the E3 ubiquitin ligase IDF1 [16,24]. IDF1 elongates monoubiquitin moieties into K63 polyubiquitin chains, thus leading to IRT1 targeting the vacuole for degradation [16,24]. Fe^2+^ availability has, however, no influence on IRT1 protein localization since IRT1 is not specifically degraded upon iron resupply under physiological metal conditions [16,24].

The cytosolic regulatory loop of IRT1 located between transmembrane domains TM4 and TM5 (amino acid 144–185) has been shown to bind metals *in vitro* [24]. A remarkable feature of IRT1 and related transporters is the presence of a histidine-rich motif at the center of such loop. Such repetition of histidine has been shown to be required for the non-iron metal-dependent endocytosis of IRT1, as an IRT1_4HA_ mutant with the four histidines mutated to alanine fails to be degraded upon non-iron metal excess [24]. Metal binding to histidine residues was shown to drive the recruitment of CIPK23 [24]. IRT1 is, therefore, considered a bifunctional transporter–receptor capable of sensing elevated non-iron metal levels and initiating a signaling cascade culminating in its self-degradation to limit the entry of highly reactive non-iron metals. The intricate mechanism allowing metal binding to IRT1 histidine-rich motif to trigger CIPK23 recruitment remains, however, still unclear but was proposed to involve the folding of IRT1 [25].

To obtain better mechanistic insight into how IRT1 senses non-iron metals and how this mediates the recruitment of CIPK23, we biochemically characterized the metal-binding properties and the structure of the IRT1 loop by a combination of circular dichroism (CD), NMR spectroscopy, and microscale thermophoresis (MST). We report that the regulatory loop of IRT1 binds Zn^2+^ and Mn^2+^ *in vitro* using the four histidine residues with nanomolar affinities, and we also uncover the role of residue D173 in Zn^2+^ and Mn^2+^ co-ordination and sensing during IRT1 degradation in response to non-iron metal excess. Furthermore, we show that the regulatory loop of IRT1 is disordered in both the absence and presence of Zn^2+^, despite experiencing small structural changes in the presence of Zn^2+^. Overall, our work provides additional biochemical and structural insight into the ZIP family of metal transporters.

## Results

### The regulatory loop of IRT1 is disordered

**Figure 1:**
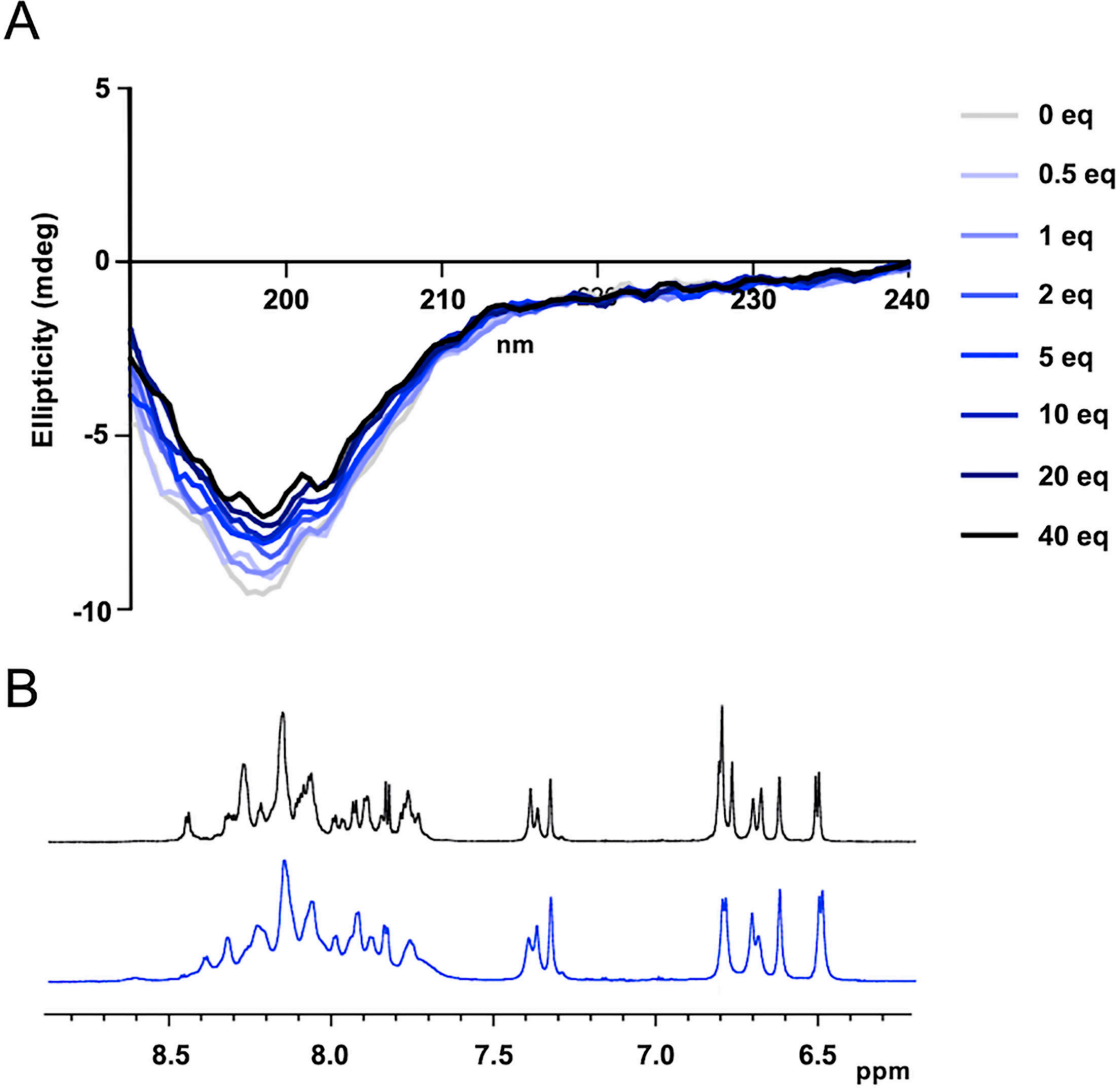
The IRT1 regulatory loop is disordered. (**A**) Far-UV circular dichroism spectra (CD) of the IRT1 regulatory loop. Equivalents of Zn^2+^ (0–40 eq) were added to IRT1 peptide, and CD spectra were recorded from 190 to 240 nm. Corrections of the final peptide concentrations were done with each addition of Zn^2+^. The data presented are an average of 128 scans. (**B**) One-dimensional (1D) ^1^H NMR spectra of the IRT1 loop recorded in the absence (black) and presence (blue) of Zn^2+^. Only the amide and the aromatic protons are shown. IRT1, iron-regulated transporter 1.

Considering that, to date, eukaryotic ZIP transporters cannot be expressed and purified from heterologous systems, we focused on the regulatory loop of IRT1 that carries important residues for metal sensing and response [24], as previously done for hZIP4 [26,27]. To get a first glimpse of the structural features of the regulatory loop of IRT1 (a.a. 144–185), we performed far-UV CD on the corresponding peptide chemically synthesized. The ^143^DSMATSLYTSKNAVGIMPHGHGHGHGPANDVTLPIKEDDSSN^186^ peptide sequence encompasses all important residues required for phosphorylation and ubiquitination, and contains the four histidine residues that have been previously associated with metal sensing [16,24]. Structure prediction using the PONDR (http://www.pondr.com) software suggests a high degree of disorder in this region (Supplementary Figure S1), with 45% of the residues being disorder promoting (A, R, G, Q, S, P, E, and K), and only 21% being rather order promoting (W, C, F, I, Y, V, L, and N). In accordance with software predictions, CD spectra recorded from 190 to 240 nm at pH 6.7 at room temperature showed a spectrum typical of a peptide in a random coil conformation, with a single negative peak at 198 nm [28] (Figure 1A, gray line, 0 eq).

**Figure 2:**
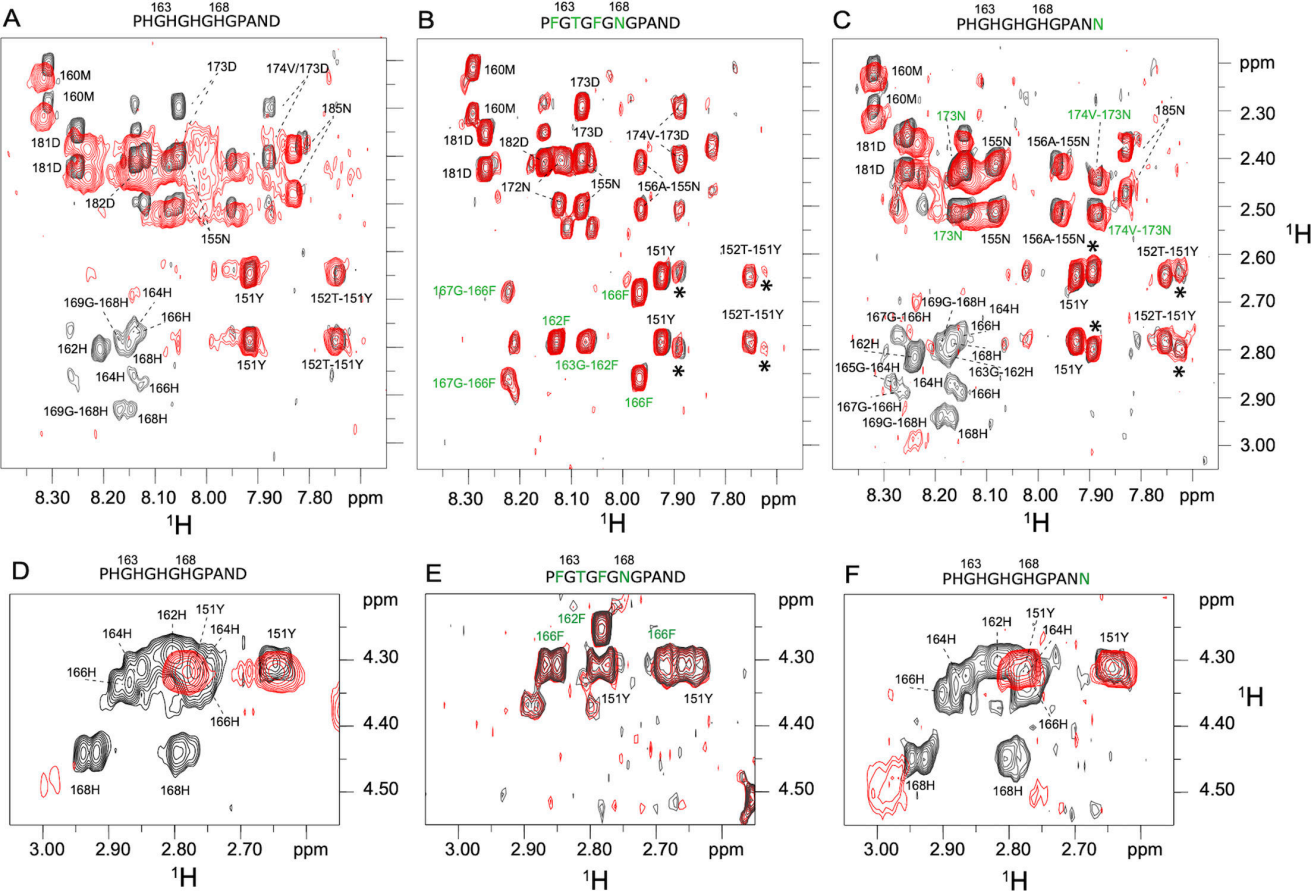
Zinc directly binds to histidine residues in the IRT1 regulatory loop. Fingerprint region of the ^1^H-1H NOESY spectrum recorded on the IRT1 loop in the presence or absence of Zn^2+^. Several intramolecular and intermolecular NH-CHβ signals are shown for the wildtype IRT1 loop (**A**) and two mutant peptides (**B,C**); and intramolecular CHα-CHβ signals for the wildtype IRT1 loop (**D**) and two mutant peptides (**E, F**). The sequences of the domain into which mutations have been introduced are shown above each spectrum, and the mutations are highlighted in green. The spectra recorded in the absence of Zn^2+^ are shown in black, and those recorded in the presence of two molar equivalents of Zn^2+^ are shown in red. Asterisks (*) indicate impurities in the sample. Annotations with a dash indicate cross-peak between inter-residue, that is NHi-CHβi-1, and without dash cross-peaks between intra-residue, that is HNi-Hβi. IRT1, iron-regulated transporter 1.

To obtain deeper insight into the structure of the IRT1 loop, we turned to NMR spectroscopy. One-dimensional (1D) ^1^H NMR spectra were recorded at pH 6.7 in the absence of metals. In line with the results obtained by CD, 1D ^1^H NMR spectra were poorly dispersed, a characteristic of peptides in random coil conformations (Figure 1B, black line). Furthermore, two-dimensional (2D) H^1^-H^1^ NOESY spectra of the IRT1 loop showed that despite our previous observations of the peptide adopting a rather unstructured conformation, medium-range nuclear Overhauser effect (NOE) existed, notably pointing to the presence of a turn involving the ^155^AVGI^160^ hydrophobic residues (α156/HN159, α157/HN159) (Supplementary Figure S2A). Besides, we observed that the cross-peak intensities of the histidine-rich domain from IRT1 decreased relative to the other signals of the peptide, resulting probably from chemical exchange (Figure 2A). Despite the lack of a clear secondary structure, we generated structures using the NOE-derived distance restraints with the CYANA software (Supplementary Figure S2). As expected, except for the ^155^AVGI^160^ turn (Supplementary Figure S2), no secondary structure was observed.

### Impact of metal binding on the structure of the IRT1 regulatory loop

In order to determine whether the structural properties of the IRT1 loop are affected by interaction with the secondary metal substrates of IRT1, we performed a titration by sequential equimolar additions of Zn^2+^ on the peptide followed by recording of CD spectra (Figure 1A). With each addition of Zn^2+^, the final concentration of the peptide was corrected to avoid dilution effects. We observed a saturable increase in ellipticity with each addition of the substrate, but the overall shape of the spectra remained unchanged. Overall, the binding of Zn^2+^ did not seem to affect the structure of the IRT1 loop, as visualized by CD. Similarly, 1D H^1^ NMR spectra in the presence of Zn^2+^ showed a similar dispersion of the signals compared with the spectra recorded in the absence of metal, again suggesting that even in the presence of Zn^2+^, the IRT1 loop remains disordered (Figure 1B, blue line). Interestingly, the signals are broadened upon sequential addition of Zn^2+^, indicating that the IRT1 loop undergoes small structural changes probably induced by the binding of Zn^2+^ ions (Figure 1A, blue-colored lines).

To obtain a deeper understanding of the molecular interaction of the IRT1 loop with its substrates, 2D H^1^-H^1^ NOESY spectra of the IRT1 peptide were compared at pH 6.7 in the absence of metal substrates and in the presence of two equivalents of Zn^2+^. Superimposition of these spectra indicated that the histidine-rich motif of IRT1 is implicated in the binding of Zn^2+^ since all the proton resonances of these histidines (H162, H164, H166, and H168) disappeared upon the addition of Zn^2+^ (Figure 2A). This suggests that these residues are involved in the exchange between several conformations in an intermediate regime. Further analysis of our data also indicated that signals corresponding to D173, which is located C-terminal of the histidine-rich stretch, behaved similarly to the histidine residues (Figure 2A). NMR signals corresponding to D173 indeed disappeared after the addition of Zn^2+^, suggesting a possible implication of D173 in metal co-ordination.

To better characterize the role of individual histidine residues and aspartic acid D173 present in the wildtype IRT1 loop, we also decided to record H^1^-H^1^ NOESY spectra of different mutant peptides. We designed three mutants for the PHGHGHGHGPAND motif where histidine residues were replaced with phenylalanine (F), threonine (T), or asparagine (N) to remove the charges at these positions and maintain closest residue similarity; and a mutant where aspartic acid D173 was replaced by the uncharged asparagine amino acid. We observed that in the presence of Zn^2+^, the line width for most of the peptides increased drastically (Figure 2A,C,D,F and Supplementary Figure S3) except for the mutant peptide with four mutated histidine residues (Figure 2B and E). Broadening of the signals indicates that the metal-bound IRT1 loop undergoes dynamic interchange among different metal-bound states. Interestingly, the mutant peptides with only the first two (H162 and H164) or last two histidine residues (H166 and H168) from the stretch mutated to uncharged residues were still able to bind Zn^2+^ (Supplementary Figure S3). Furthermore, the signal for the D173N mutant peptide no longer responded to the addition of Zn^2+^ (Figure 2C and F), suggesting that D173 is indeed involved in metal co-ordination.

**Figure 3:**
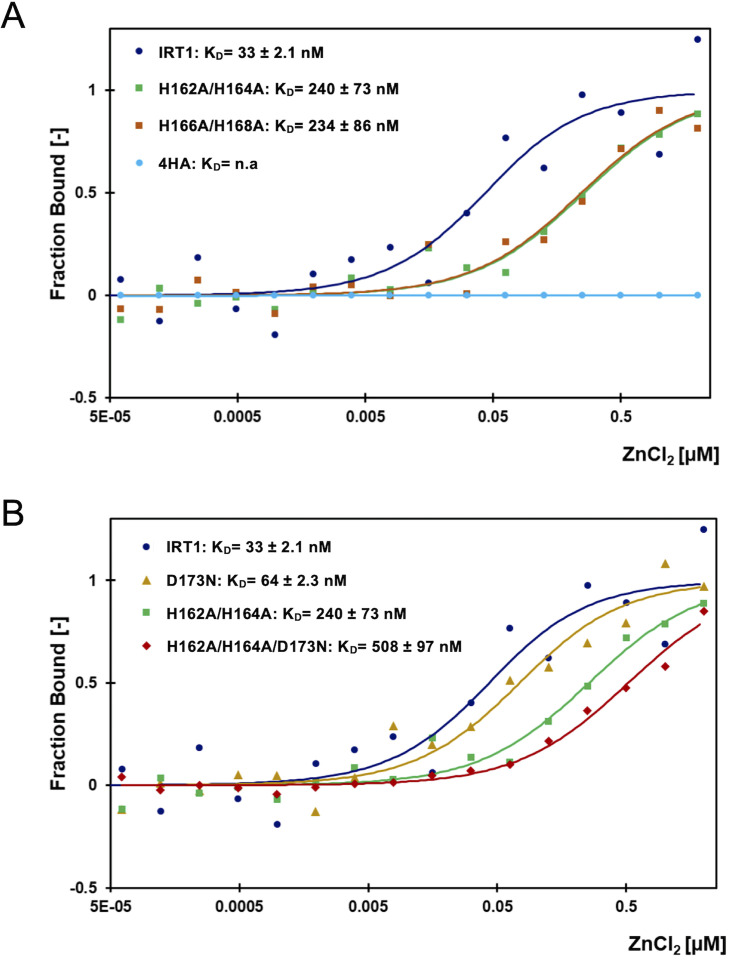
Zinc binding affinities of the IRT1 regulatory loop. (**A**) Microscale thermophoresis (MST) analyses of zinc binding to the wildtype IRT1 loop (IRT1; dark blue), double mutant with histidine residues 162 and 164 mutated to alanine (H162A/H164A; green), double mutant with histidine residues 166 and 168 mutated to alanine (H166A/H168A; orange), and quadruple mutant with histidine residues 162, 164, 166, and 168 mutated to alanine (4HA; light blue). Dots represent the average dose response of at least six technical replicates derived from two biological replicates. Errors bars and MST binding parameters are shown in online supplementary figure 5. (**B**) MST analyses of zinc binding to the wildtype IRT1 loop (IRT1; dark blue), single mutant with aspartic acid 173 mutated to asparagine (D173N; yellow), double mutant with histidine residues H162 and H164 mutated to alanine (H162A/H164A; green), and triple mutant with histidine residues 162 and 164 mutated to alanine and aspartic acid 173 mutated to asparagine (H166A/H168A/D173N; red). Dots represent the average dose response of at least six technical replicates derived from two biological replicates. Errors bars and MST binding parameters are shown in online supplementary figure 5. IRT1, iron-regulated transporter 1.

Finally, we analyzed the chemical shift variations in the absence and presence of two equivalents Zn^2+^ of each residue. Here, we found that the residues located at the N-terminal side of the histidine-rich fragment were very little affected by Zn^2+^ binding, whereas those located on the C-terminus underwent significant chemical shift variations (Supplementary Figure S4A). The same behavior was observed for peptides with mutations of two histidine residues and for the aspartic acid mutant (Supplementary Figure S4B-D), though to a lesser extent than for the wildtype peptide. As expected, the peptide with the four histidine residues mutated presented no chemical shift variation (Supplementary Figure S4E).

These results are in line with the amino acid composition of the IRT1 loop, which is rich in negatively charged amino acids (E and D) on its C-terminal side with an isoelectric point of 3.84 (a.a. 168–185), instead of 5.83 for its N-terminal side (a.a. 144–167).

### Metal-binding affinities of the IRT1 regulatory loop

In order to quantify more precisely the interaction between the regulatory loop of IRT1 and non-iron metals, we performed MST experiments. We first used Zn^2+^ as substrate, as we previously showed that it binds to the IRT1 peptide by ICP-MS [24], MST [16], and CD (this study), and Zn^2+^ excess was reported to drive IRT1 endocytosis [24]. A constant concentration of the peptide was titrated with a one in half-serial dilution of the ligand, ZnCl_2_, and thermophoresis was measured. A binding affinity of 33 ± 2.1 nM, when fitted to the *Kd* model, was obtained for the wildtype IRT1 loop assuming a 1:1 stoichiometry of the reaction (Figure 3A). Data points from the peptide mutated for the four histidine residues (4 HA) were recorded and failed to fit a binding curve, confirming the absolute requirement of the histidine stretch for metal binding (Figure 3A) [16]. To evaluate deeper the contribution of the residues from the histidine stretch, we decided to test double mutant peptides harboring H162A/H164A or H166A/H168A mutations. Interestingly, both double histidine substitutions resulted in reduced affinities for Zn^2+^ compared with wildtype, with a *Kd* of 240 ± 73 nM for the H162A/H164A mutant and a *Kd* of 234 ± 86 nM for the H166A/H168A mutant (Figure 3A).

To inquire about the role of the D173 residue in metal-binding affinity, the IRT1 loop carrying the D173N mutation was subjected to MST analyses. Binding curves for Zn^2+^ were determined in the same concentration range as the previous experiments and fitted to the *Kd* model. The binding curve obtained for the D173N mutant was slightly shifted to the right compared with the one obtained with the wildtype peptide (Figure 3B), with a *Kd* of 64 ± 2.3 nM (Figure 3B). Because the contribution of D173 is likely minor compared with histidine residues, we sought to investigate the contribution of D173 in a peptide carrying a double histidine mutation where Zn^2+^ co-ordination is already destabilized, as shown above. The peptide carrying the three mutations showed significantly lower affinity to Zn^2+^ compared with the peptide with only two histidine residues mutated (Figure 3B). Such decrease in the affinity due to the removal of the negative charge from D173, in the context of the H162A/H164A mutation, argues for a contribution of D173 to Zn^2+^ co-ordination during sensing by the IRT1 transceptor.

**Figure 4:**
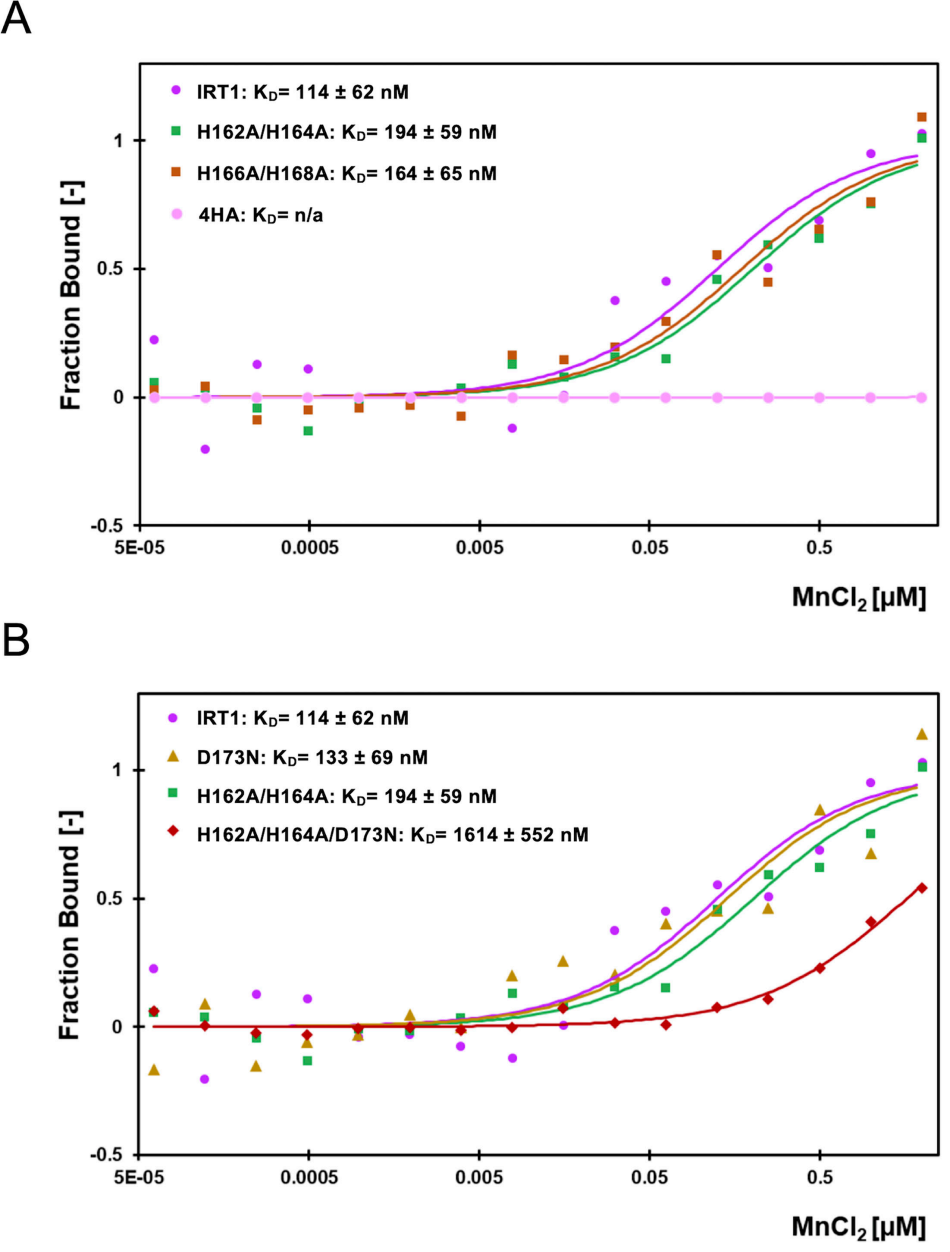
Manganese binding affinities of the IRT1 regulatory loop. (**A**) Microscale thermophoresis (MST) analyses of manganese binding to the wildtype IRT1 loop (IRT1; dark pink), double mutant with histidine residues 162 and 164 mutated to alanine (H162A/H164A; green), double mutant with histidine residues 166 and 168 mutated to alanine (H166A/H168A; orange), and quadruple mutant with histidine residues 162, 164, 166, and 168 mutated to alanine (4HA; light pink). Dots represent the average dose response of at least six technical replicates derived from two biological replicates. Errors bars and MST binding parameters are shown in online supplementary figure 6 . (**B**) MST analyses of manganese binding to the wildtype IRT1 loop (IRT1; dark pink), single mutant with aspartic acid 173 mutated to asparagine (D173N; yellow), double mutant with histidine residues H162 and H164 mutated to alanine (H162A/H164A; green), and triple mutant with histidine residues 162 and 164 mutated to alanine and aspartic acid 173 mutated to asparagine (H162A/H164A/D173N; red). Dots represent the average dose response of at least six technical replicates derived from two biological replicates. Errors bars and MST binding parameters are shown in online supplementary figure 6 . IRT1, iron-regulated transporter 1.

Considering that IRT1 also transports Mn^2+^ and that this non-iron metal was also shown to regulate IRT1 degradation [8,10,11,14,15,24], we monitored Mn^2+^ binding to the IRT1 loop by MST. We observed that the IRT1 loop is also able to bind Mn^2+^ at nanomolar range, with lower affinity than for Zn^2+^, showing a *Kd* of 114 ± 62 nM (Figure 4A). Similar to what has been observed with Zn^2+^, the four histidine IRT1 mutant loop was no longer able to bind Mn^2+^ and the double histidine mutants presented lower affinity compared with wildtype IRT1. Interestingly, the absence of two histidine residues showed less impact on the affinity for Mn^2+^ (Figure 4A) compared with Zn^2+^ (Figure 3A), pointing to the possibility of others residues being actively involved in Mn^2+^ co-ordination. To test whether D173 could also participate to Mn^2+^ binding, we studied the affinity of the mutants harboring D173N mutation. Single D173N mutation barely affected affinity for Mn^2+^ compared with wildtype (Figure 4B). Nevertheless, D173N combined with H162A/H164A mutations resulted in a strong decrease in Mn^2+^ affinity (Figure 4B), also revealing a strong contribution of these three residues to Mn^2+^ co-ordination.

### Analysis of the role of aspartic acid 173 in IRT1 response to metal excess

To characterize the role of residue D173 in metal transport and sensing, we first decided to express full-length IRT1_D173N_ in the iron uptake-defective *fet3fet4* yeast mutant to evaluate whether the corresponding protein is still active for iron transport. Yeast expressing wildtype IRT1 were able to complement the growth defect of *fet3fet4* in low-iron conditions, as previously reported [10]. Expression of IRT1_D173N_ protein yielded comparable growth compared with wildtype IRT1, indicating that IRT1_D173N_ is fully functional for iron transport (online supplementary figure 7A).

**Figure 5:**
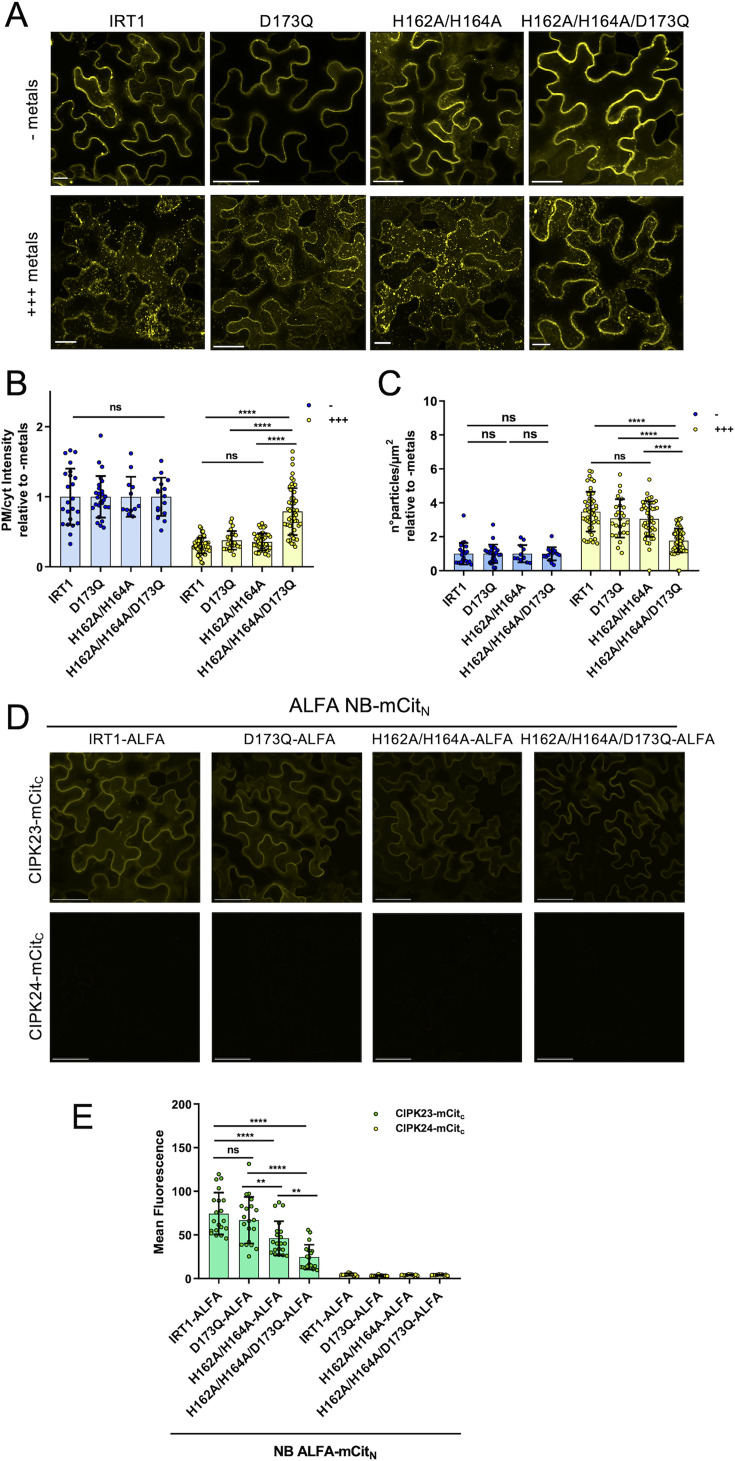
Residue D173 is involved in IRT1 endocytosis in response to non-iron metal excess. (**A**) Representative confocal microscopy images of epidermal cells from *Nicotiana benthamiana* leaves transiently expressing 35::IRT1-mCitrine (IRT1) or mutated versions 35S::IRT1_D173Q_-mCitrine, 35S::IRT1_H162A/H164A_-mCitrine (H162A/H164A), and 35S::IRT1_H162A/H164A/D173Q_-mCitrine (H162A/H164A/D173Q) after 3 h of control treatment (without non-iron metals; − metals) or after non-iron metal excess treatment (+ ++ metals). Showed maximum projection of 10–15 optical sections taken using 1 µm z-distance. Scale bars, 20 μm. (**B**) Quantification of the ratio of the plasma membrane to intracellular signals and (**C**) quantification of intracellular particles per µm^2^ from cells exposed to metal excess relative to cells exposed to control solution of plants treated as described in A). Error bars represent SD (*n* = 25–50) from three independent experiments. ‘ns’ indicate no significant differences, and asterisks indicate significant differences (one-way ANOVA, Tukey post-test, ****P<0.0001). (**D**) Trimolecular fluorescence complementation (TriFC) assay with CIPK23 fused to the mCit_C_ fragment and ALFA tag-bearing IRT1 variants (from left to right, IRT1, D173Q, H162A/H164A, and H62A/H64A/D173Q) co-expressed with ALFA-NB C-terminally fused to the mCit_N_ fragment. CIPK24, which does not interact with IRT1, is used as negative control. Representative images of two biological replicates are shown. Scale bars, 10 µm. (**E**) Quantification of the reconstituted mCitrine signal upon association of CIPK23 or CIPK24 to IRT1, IRT1_D173Q_, IRT1_H162A/H164A_, or IRT1_H162A/H164A/D173Q_. ‘ns’ indicate no significant differences, and asterisks indicate significant differences (one-way ANOVA, Tukey post-test, ****P<0.0001). IRT1, iron-regulated transporter 1.

An excess of non-iron metal substrates of IRT1 (Zn^2+^, Mn^2+^) leads to IRT1 depletion from plasma membrane and increases IRT1 accumulation in endosomes for later degradation in the lytic vacuole [24]. This safety mechanism to limit heavy metal toxicity in plants due to metal overaccumulation through IRT1 involves metal sensing by the histidine-rich motif located in the regulatory loop of IRT1 [16,24]. Considering that the combination of D173N and H162A/H164A mutations impairs the IRT1 loop affinity for Zn^2+^ and Mn^2+^ to a greater extent than the H162A/H164A mutation (Figures 3B and 4B), we wondered if this aspartic acid also participates to metal sensing and metal excess-dependent degradation of IRT1 protein. To test this hypothesis, we investigated the functional impact of the sole D173 mutation on IRT1 degradation in response to non-iron metal excess *in planta*. For this purpose, we took advantage of the previously reported fluorescently tagged version of IRT1, IRT1-mCitrine, which has been demonstrated to complement the *irt1-1* knockout Arabidopsis plants and *fet3fet4* [24]. IRT1-mCitrine variants were transiently expressed in *Nicotiana benthamiana* leaves under the control of the 35S constitutive promoter to study IRT1 localization in response to high levels of non-iron metals. We, however, observed that the D173N substitution yielded retention of IRT1 into intracellular structures resembling the endoplasmic reticulum (ER) (online supplementary figure 8A). This is likely explained by the unexpected generation of an N-glycosylation site at the corresponding position (online supplementary figure 8B). We, therefore, substituted the D173 residue with glutamine (Q) to prevent spurious N-glycosylation and ER retention IRT1 mutated at D173 residue (online supplementary figure 8B). As expected, IRT1_D173Q_ localized at the plasma membrane similarly to wildtype IRT1 (online supplementary figure 8A). IRT1-mCitrine was observed mostly at the plasma membrane when expressed in *N. benthamiana* in the absence of non-iron metals (− metals; Figure 5A), similar to what has been reported in stable Arabidopsis transgenic plants [15,16,24,29]. A 3-h treatment with non-iron metal excess (+++ metals) led to IRT1-mCitrine depletion from plasma membrane and internalization in endosomes (Figure 5A and B), as already observed in roots [24]. IRT1 variants harboring the D173Q single mutation and the H162A/H164A double mutation exposed to a non-iron metal excess also displayed reduced plasma membrane localization (Figure 5A and B) and an increased number of endosomes (Figure 5C). Consistently, the interaction of the corresponding IRT1 variants with CIPK23 measured by trimolecular functional complementation (TriFC) is not (IRT1_D173Q_) or marginally (IRT1_H162A/H164A_) affected (Figure 5D and E). Interestingly, the triple mutant for H162A/H164A/D173Q showed significantly less protein removal from the plasma membrane (Figure 5A and B) and a significantly lower number of endosomes compared with double mutant H162A/H164A after treatment with high non-iron metals (Figure 5C). Such observation is not explained by a defect of IRT1_H162A/H164A/D173Q_ to transport metals as its expression in the *fet3fet4* yeast mutant and in an Arabidopsis *irt1_crispr_* mutant complemented growth to the same extent as IRT1 and IRT1_H162A/H164A_ (online supplementary figure 7B and C). The reduced metal-dependent endocytosis of IRT1_H162A/H164A/D173Q_ is accompanied by a lower ability to interact with CIPK23 compared with IRT1 carrying only the H162A/H164A mutations (Figure 5D and E). These observations clearly argue for an implication of D173 residue in Zn^2+^ and Mn^2+^ sensing and proper IRT1 degradation when plants are experiencing non-iron metal stress. Notably, IRT1_K154R/K179R_ double mutant, which is defective in endocytosis due to its inability to be ubiquitinated, failed to be endocytosed upon metal excess (online supplementary figure 9).

Altogether, these observations indicate that *N. benthamiana* leaves possess the cellular machinery responsible for the internalization and degradation of IRT1 under metal excess and highlight the combined role of residues H162, H164, H166, H168, and D173 in co-ordinating and sensing non-iron metals to fine-tune iron and metal uptake.

## Discussion

To cope with changing nutrient availability and nutrient demand, plants developed strategies that allow them to control the expression of nutrient transporters. This is the case for the Arabidopsis IRT1 iron transporter that is controlled by various metal substrates at different levels, that is transcriptional regulation by low Fe^2+^ and post-translational regulation by Zn^2+^ and Mn^2+^ excess [24,29,30]. Previously, we have shown that the post-translational regulation of IRT1 at the plasma membrane in response to non-iron metal excess involves its phosphorylation by the CIPK23 kinase followed by decoration with K63 polyubiquitin chains by the IDF1 E3 ligase, mechanism that requires the histidine-rich motif in the IRT1 regulatory loop [24].

To gain further insight into the molecular mechanisms of metal sensing by IRT1, we here investigate the structural basis of the IRT1 regulatory loop using a combination of NMR spectroscopy, CD spectroscopy, MST analyses, and protein prediction algorithms. We show that the IRT1 loop is an intrinsically disordered region (IDR), which can adopt various conformations. This characteristic appears to be conserved among ZIPs, as it is also observed for the human ZIP4 zinc transporter [26,27]. This is, however, not a specific feature of ZIPs since other families of transporters, like the Arabidopsis MTP1 vacuolar zinc transporter, also possess disordered intracellular loops with metal-binding motifs [31]. Our observations also indicate that the IRT1 loop undergoes small structural changes in the presence of Zn^2+^, although it remains largely disordered. This observation points to the formation of a ‘fuzzy’ complex between an IDR and a small ligand, as previously reported for hZIP4 [27]. Intrinsically disordered proteins (IDPs) or IDR interactions can exhibit varying mechanisms, ranging from complete folding upon binding to the formation of such 'fuzzy' complexes. The term 'fuzzy complex' refers to a class of interactions in which the involved IDPs or IDRs retain conformational flexibility upon binding. Unlike traditional stable protein complexes, these interactions are characterized by dynamic behavior, with the complexes not adopting a single, fixed conformation. The enhanced flexibility and conformational plasticity of IDRs in proteins provide a platform for post-translational events allowing enhanced interactions and chemical reactions between partners [32,33]. For IRT1, such capacity may facilitate the recruitment of downstream factors such as the CIPK23 kinase for the IRT1 loop phosphorylation upon non-iron metal excess [24]. Moreover, the importance of the histidine stretch of the IRT1 regulatory loop in its post-translational regulation has been previously established, where plants harboring a protein mutated for the four histidine residues fail to show IRT1 phosphorylation and degradation upon non-iron metal excess [24]. Interestingly, we observed chemical shift variations in the residues located at the C-terminus of the regulatory loop in the presence of Zn^2+^ compared with its absence, a region where the predicted phosphorylated T175 by CIPK23 is found [24]. Nevertheless, because the histidine resonances disappear upon the addition of Zn^2+^, we are not able to determine whether this particular stretch adopts a specific conformation in such conditions.

The nanomolar range dissociation constants recorded for Zn^2+^ and Mn^2+^ binding to IRT1 are consistent with experimentally determined nanomolar concentrations of such metals in plants grown in excess conditions [34]. Importantly, we unambiguously reveal that the histidine stretch is of absolute importance for direct Zn^2+^ and Mn^2+^ co-ordination, as MST experiments demonstrated intense perturbations upon mutation of all four histidines, completely abolishing metal binding. Surprisingly, we uncovered that the IRT1 loop is still able to bind Zn^2+^ when two histidine residues are mutated, probably due to the other two histidines and aspartic acid 173 being responsible for co-ordination. This is supported by the NMR data obtained with the double histidine mutants where proton resonance of the remaining two histidine residues and aspartic acid 173 still disappeared in the presence of Zn^2+^. Although a co-ordination number of three for Zn^2+^ is not common, Zn^2+^ is described to adopt a variety of distorted co-ordination geometries without significant energy penalty [35]. Besides, we speculate that the IRT1 loop flexibility, as an IDR, could explain the remaining ability of triple mutant H162A/H164A/D173N to bind Zn^2+^, by allowing surrounding residues contribute to Zn^2+^ co-ordination, as predicted by Aphafold server 3 [36] for aspartic acid residue 144 (online supplementary figure 10).

**Figure 6:**
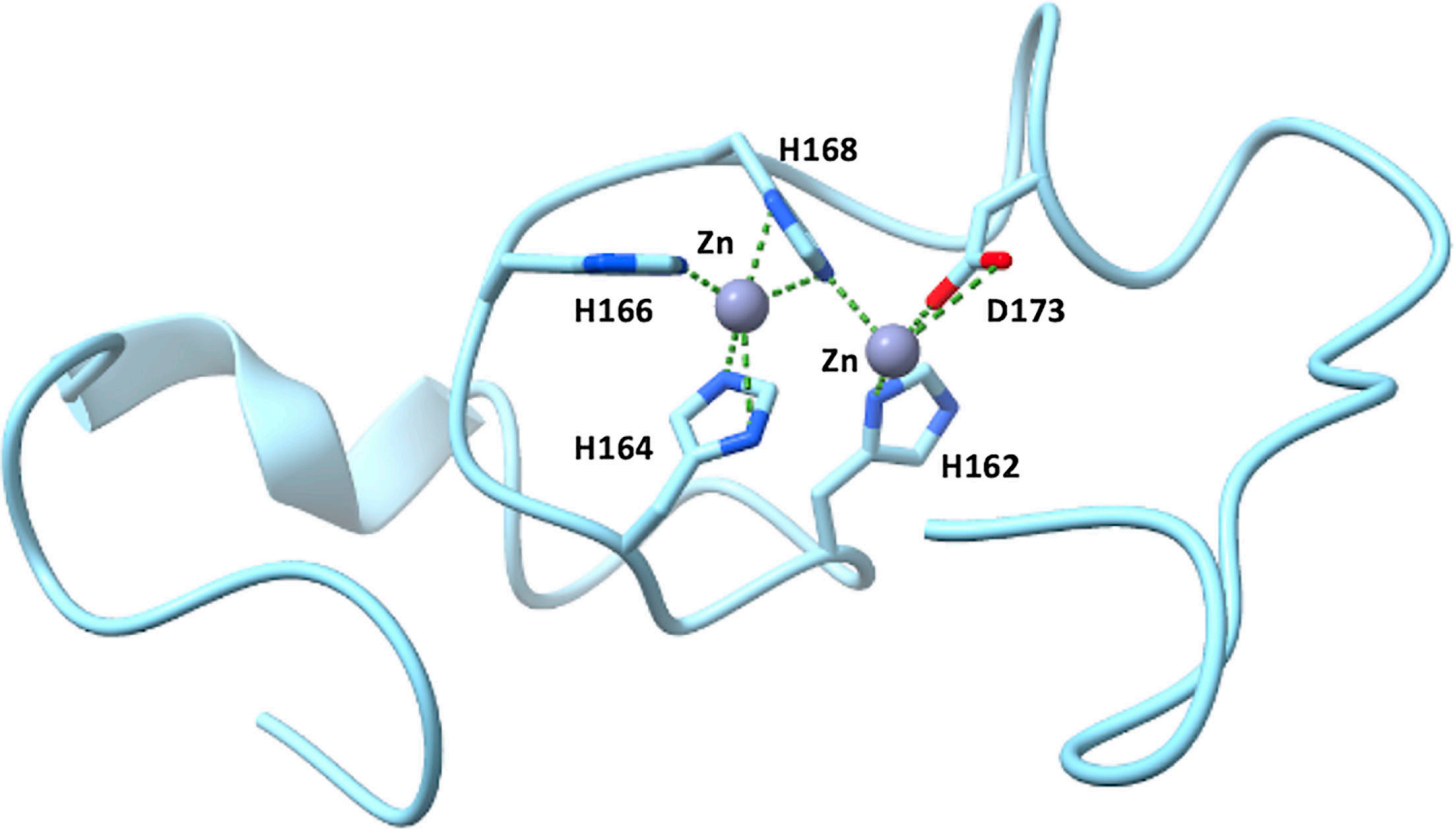
Zinc and manganese ion co-ordination by the IRT1 regulatory loop. Alphafold3 prediction for the IRT1 loop co-ordinating Zn^2+^ ions (gray spheres) with residues H162, H164, H166, H168, and D173. Slashed green lines represent metal co-ordination within 3.5 Å distance. Analyses performed with UScf. ChimeraX application. IRT1, iron-regulated transporter 1.

Based on our NMR and MST observations, five residues from the regulatory loop of IRT1 could co-ordinate Zn^2+^. Since two Zn^2+^ ions have already been described to be co-ordinated by five ligands in proteins with one of the ligands would be acting as a bridge [37], two Zn^2+^-binding sites may also exist in the IRT1 loop. This hypothesis is also supported by prediction made using Alphafold server 3 for the IRT1 loop in the presence of two Zn^2+^ ions, where histidine 168 is presented as the bridge co-ordinating both ions (Figure 6). Unfortunately, the possible existence of two Zn^2+^-binding sites in the IRT1 loop cannot be distinguished with our data. As postulated for the histidine-rich cytosolic loop of hZIP4 [27], we claim that a single Zn^2+^-bound state probably does not exist, but rather the Zn^2+^-bound state is likely a conformational ensemble with the Zn^2+^ co-ordinated by multiple combinations of the histidine residues and aspartic acid 173, allowing transient Zn^2+^-binding modes within IRT1 depending on cytosolic Zn^2+^ concentrations. We also uncovered that the same residues involved in Zn^2+^ binding also participate to Mn^2+^ co-ordination, probably along with neighboring residues. We, however, demonstrate here that the IRT1 loop shows higher affinity to Zn^2+^ compared with Mn^2+^, consistent with the stronger degradation of IRT1 observed in plants facing Zn excess [24]. Metal ion availability and relative local cytoplasmic concentrations will likely dictate whether the IRT1 loop will be co-ordinating Zn^2+^, Mn^2+^, or both.

Altogether, our work offers a framework for the analysis of metal-sensing properties of ZIP transporters and deepens our understanding of how IRT1 protein senses metals through its regulatory loop. This understanding is crucial to grasp how plants optimize iron uptake and limit the absorption of highly reactive non-iron metals in plant tissues and to consider biotechnological approaches to modulate heavy metal accumulation in plants.

## Experimental procedures

### Circular dichroism

CD spectra were recorded on a Jasco J-815 spectropolarimeter equipped with a temperature controller operating at room temperature. CD spectra ranging from 190 to 240  nm were recorded in a 1-mm path length cuvette. Sample concentrations were at 25 µM, in 10 mM Tris, pH 7.0, and the data presented are an average of 128 scans.

### Sample preparation and NMR experiments

The wildtype IRT1 loop peptide and the four mutant peptides were synthesized by Proteogenix with a purity grade of 95% minimum. Peptides were first dissolved at pH 3.5 to a final concentration of 500 µM in the presence of 150 mM NaCl. The pH was then adjusted to 6.7. Then, two equivalents of ZnCl_2_ for the peptide concentrations were added, and the pH was again adjusted to 6.7. A 2D phase-sensitive 1H Clean-TOCSY [38] with 60 ms spin lock and NOESY experiments [38] with 200 ms mixing time were recorded at 5°C and 20°C on an AVANCE Bruker 800.13 MHz spectrometer, with a spectral width of 8013 Hz, without sample spinning, with 2 k real points in t2 and 512 t1-increments. Pulsed-field gradient-based WATERGATE was used for water suppression [39]. Three independent samples were prepared to test different conditions, such as salt concentration, pH, and temperature. The data were processed using TopSpin 4.0.6 software (Bruker). π/3 and π /6 phase-shifted sine bell window function was applied before Fourier transformation in both dimensions (t1 and t2). Data processing and analysis were performed using the Topspin ® 4.0 and CCPN NMR software [40].

### NMR structure of the IRT1 loop

Interproton distance restraints were derived from the 2D ^1^H NOESY (with a 200 ms mixing time and at 5**°**C) using CcpNmr 2.4 [40] and used to generate the IRT1 loop structures with the program CYANA version 3.98.5 [41]. We used the standard CYANA protocol of seven iterative cycles of NOE assignment and structure calculation, followed by a final structure calculation. In each cycle, the structure calculation started from 200 randomized conformers, and the standard CYANA simulated annealing schedule was used with 10,000 torsion angle dynamics steps per conformer. Graphic representations were prepared with PyMOL [42].

### Cloning expression and protein purification for MST

The wildtype IRT1 loop (a.a. 144–185) was cloned in the pMalc2x vector, in frame with the sequence encoding the maltose-binding protein (MBP) tag. H162A, H164A, H166A, H168A, and D173N mutations were introduced on the pMal-IRT1 vector by primer reactions in parallel SPRINP method [43], using primers listed in Supplementary Table S1. The different constructs generated are found in Supplementary Table S2. Vectors were transformed into BL21 DE3 *E. coli,* and recombinant proteins were purified using the manufacturer’s recommendation. Briefly, 1l of LB supplemented with glucose at 0.2% (w/v) final concentration, and ampicillin was inoculated with 10 ml of an overnight high-density culture of cells containing the fusion plasmids pMal containing wildtype and mutants IRT1. Cultures were grown at 37°C upon agitation until OD_600_ reached 0.5. The expression of the wildtype and the IRT1 loop mutants was induced by the addition of IPTG to a final concentration of 0.3 mM, and cell cultures were incubated at 20°C overnight. Pellets were recovered by centrifugation at 10,000×g for 40 min at 4°C, then suspended in HEPES 10 mM pH 7, NaCl 150 mM in 30 ml/l culture. Suspended cells were stored until further processing at −80°C.

Affinity purification of the MBP-IRT1 loop variants was performed in batches. Frozen cell suspensions were thawed. Lysozyme was added for cell wall disruption, and then the suspensions were sonicated with eight pulses for 45 seconds while keeping on ice. Clarification of the lysate was performed at 20,000×g for 30 min. The supernatant (crude extract) was recovered and stored in ice. Separately, 500 µl of amylose resin (NEB) was washed according to the provider’s specifications. Washed amylose resin was incubated with the crude extract in rotation at 4°C for 1 h 30 min. The resin was then pelleted at 500 g, and flow through was discarded. Two washing steps were performed with HEPES 10 mM pH 7, NaCl 150 mM, 1 mM EDTA, followed by two more washes in the same buffer without EDTA. Three consecutive elutions were carried out with HEPES 10 mM pH 7, NaCl 150 mM, and Maltose 10 mM.

### Microscale thermophoresis

Binding experiments were performed by MST with a Monolith NT.115 (NanoTemper® Technologies, Munich, Germany). The MBP-IRT1 loop variants were labeled with the Monolith NT™ Protein Labeling Kit RED according to the instructions provided by the manufacturer, using a 1:3 protein:dye molar ratio. For binding experiments, the labeled proteins (20 nM) were incubated with a range of titrant concentrations made by serial dilutions (1:2), in 50 mM Tris buffer pH 7.4, 10 mM MgCl_2_, 150 mM NaCl, 0.05% Tween 20, in PCR tubes, at room temperature for 10 min. Premium treated capillaries (NanoTemper® Technologies) were loaded and the measurements were performed at 25°C, 40% LED power and 40% MST power, 20 s laser‐on time and 1 s laser‐off time. All the experiments were repeated three times with two independent protein-labeling reactions. Binding data were analyzed using MO. Affinity Analysis software (NanoTemper® Technologies).

### IRT1 constructs for plant and yeast expression

IRT1 mutations H162A, H164A, and D173Q were introduced into the pDONR-IRT1mCitrine [24] or the pDONR-IRT1-ALFA vectors [44] using the SPRINP method [43], using primers listed inSupplementary Table S1. For plant expression, final destination vectors were obtained by MultiSite Gateway® recombination using the entry vector described above, the pDONR-P4P1R-p35S entry vector containing the 2 × 35S promoter sequence, the pDONRP2RP3 entry vector containing a mock sequence, and the pGm43GW destination plasmid for expression in plants [24,45,46]. For yeast expression, final destination vectors were obtained by MultiSite Gateway® recombination using the entry pDONR-IRT1mCitrine vector harboring the mutations of interest and pDR195 destination vector.

### Yeast complementation assay

The complementation of the wildtype yeast strain Y00000 (*Mata; leu2Δ0; ura3Δ0; met15Δ0*) and *fet3fet4* (Mata; leu2Δ0; ura3Δ0; lys2Δ0; YMR058w::kanMX4;YMR319c::kanMX4) yeast mutant was performed by expressing IRT1 or IRT1 mutated versions (IRT1_D173N_, IRT1_D173Q_, IRT1_H162A/H164A_, or IRT1_H162A/H164A/D173Q_) in the pDR195 yeast expression vector. Transformants were selected on a selective medium lacking uracil. For complementation assays, strains were grown at 28°C for three or four days on a selective medium without iron or containing 100 µM of Fe-EDTA.

### Arabidopsis complementation assay

To generate a CRISPR/Cas9 construct for genome editing of *A. thaliana* (Col-0), two guide RNAs (gRNA) targeting *IRT1* were cloned into the pHEE401 plasmid, as described in [47]. The sgRNA sequences were designed using CRISPR-P v2.0 [48] (Supplementary Table S1). T1 mutants were screened based on their chlorotic phenotype. Genomic DNA was extracted, and the targeted *IRT1* region was amplified by PCR using gene-specific primers flanking the gRNA target site. PCR amplicons were subjected to Sanger sequencing to confirm the mutation. Mutant plants were then backcrossed with wildtype *A. thaliana* to eliminate the transgene with the Cas9. *irt1_crispr_* Col-0 mutant was transformed with pIRT1:IRT1_H162A/H164A_-mCitrine and pIRT1:IRT1_H162A/H164A/D173Q_-mCitrine constructions by floral dip method [49]. Phenotype of transgenic lines were observed after growth in soil for 25 days.

### IRT1 localization assays in *N. benthamiana*

*N. benthamiana* plants were grown for four weeks on soil under 16 h light at 22°C and 55% humidity before infiltration with *Agrobacterium tumefaciens* GV3101 strain.

IRT1-mCitrine variants were infiltrated into *A. tumefaciens* GV3110, and IRT1 protein localization assays were performed 48 h after agroinfiltration. Leaves transiently expressing IRT1-mCitrine variants were infiltrated with the treatments of study, consisting of control solution: half-strength Murashige and Skoog (MS/2) medium [50] lacking iron and non-iron metals; or metal excess solution: MS/2 lacking iron and containing Zn^2+^ (150 µM) and Mn^2+^ (300 µM). After 2 h, leaves were re-infiltrated with correspondent treatment supplemented with 300 µM of cycloheximide for 1 h (in order to distinguish between newly IRT1 protein synthesized and endocytosed IRT1), and confocal images were taken.

### Trimolecular functional complementation

*N. benthamiana* leaves were infiltrated with IRT1-ALFA, ALFA NB-mCitN, and CIPK23-mCit_C_ vectors, as previously described [44]. Interaction between ALFA-tagged IRT1, recognized by the ALFA Nanobody-mCit_N_ fusion, and CIPK23-mCit_C_ allows reconstitution of mCit. CIPK24-mCit_C_ was used as negative control.

### Confocal microscopy

For IRT1-mCit fluorescence and TriFC imaging in *N. benthamiana*, infiltrated leaves were mounted in discs in each correspondent treatment solution, and confocal imaging was performed using a Leica TCS SP8 confocal laser scanning microscope (www.leica-microsystems.com/home/). A 25×-water-immersion objective was used to collect images at 1024 × 1024 pixel resolution. mCitrine excitation used the 514- nm laser line, and emission was collected from 525 to 580 nm. Laser intensity and detection settings were kept constant in individual sets of experiments.

### Data analyses of confocal images

Quantification of the ratio of the plasma membrane to cytosolic fluorescence signal and quantification of particles were calculated from the maximum projection of 10–15 optical sections taken using 1-µm z-distance. For the ratio, mean fluorescence was quantified using ImageJ software, and results are presented as the mean value ± standard deviation of *n* = 25–50 cell leaves from three independent experiments. Same cytoplasmic ROIs designed for measuring the ratio were used for quantifying particles using ComDet v.5.5.5 plugin from Image J software. One-way ANOVA was performed and the Tukey post-test method was applied to establish significant difference between means (*P*<0.0001). Statistical analyses were performed using GraphPad Prism version 7.00 software.

To quantify TriFC experiments, the mean mCit fluorescence intensity at the plasma membrane was quantified in a total of ten cells belonging to two different biological replicates. To do so, the ‘polygon selection’ tool from the ImageJ software was used, and ten values across the plasma membrane were measured per cell. One-way ANOVA was performed, and the Tukey post-test method was applied to establish significant difference between means (*P*<0.0001). Statistical analyses were performed using GraphPad Prism version 7.00 software.

### Bioinformatic tools

Alphafold tool [51] (https://alphafold.ebi.ac.uk/) and Alphafold3 server (https://golgi.sandbox.google.com) were used for IRT1 protein structure prediction; UCSF ChimeraX (https://www.rbvi.ucsf.edu/chimerax) for modeling IRT1 structure predictions and performing metal ion contact analyses; PONDR [52] (http://www.pondr.com) for predicting IRT1 degree of disorder; and NetNGlyc [53] (https://services.healthtech.dtu.dk/service.php?NetNGlyc-1.0) was used for prediction of N-glycosylation sites in IRT1 protein.

## Supplementary material

online supplementary material 1

Online supplementary figure 1

Online supplementary figure 2

Online supplementary figure 3

Online supplementary figure 4

Online supplementary figure 5

Online supplementary figure 6

Online supplementary figure 7

Online supplementary figure 8

Online supplementary figure 9

Online supplementary figure 10

Online supplementary table 1

Online supplementary table 2

## Data Availability

Authors agree to make any materials, data, and associated protocols available upon request.

## Abbreviations

CD: circular dichroism
ER: endoplasmic reticulum
IDP: intrinsically disordered protein
IDR: intrinsically disordered region
IRT1: iron-regulated transporter 1
MST: microscale thermophoresis

## References

[1] Waldron K.J., Rutherford J.C., Ford D., Robinson N.J (2009). Metalloproteins and metal sensing.

[2] Briat J., Fobis‐Loisy I., Grignon N., Lobréaux S., Pascal N., Savino G. (1995). Cellular and molecular aspects of iron metabolism in plants. Biol. Cell.

[3] Gratão P.L., Polle A., Lea P.J., Azevedo R.A (2005). Making the life of heavy metal-stressed plants a little easier. Funct. Plant Biol..

[4] Robinson N.J., Procter C.M., Connolly E.L., Guerinot M.L (1999). A ferric-chelate reductase for iron uptake from soils. Nature.

[5] Connolly E.L., Campbell N.H., Grotz N., Prichard C.L., Guerinot M.L (2003). Overexpression of the FRO2 ferric chelate reductase confers tolerance to growth on low iron and uncovers posttranscriptional control. Plant Physiol..

[6] Wu H., Li L., Du J., Yuan Y., Cheng X., Ling H.Q (2005). Molecular and biochemical characterization of the Fe(III) chelate reductase gene family in *Arabidopsis thaliana*. Plant Cell Physiol..

[7] Santi S., Schmidt W (2009). Dissecting iron deficiency-induced proton extrusion in *Arabidopsis roots*. New Phytol..

[8] Vert G., Grotz N., Dédaldéchamp F., Gaymard F., Guerinot M.L., Briat J.F. (2002). IRT1, an *Arabidopsis* transporter essential for iron uptake from the soil and for plant growth. Plant Cell.

[9] Guerinot M.L (2000). The ZIP family of metal transporters. Biochim. Biophys. Acta.

[10] Eide D., Broderius M., Fett J., Guerinot M.L (1996). A novel iron-regulated metal transporter from plants identified by functional expression in yeast. Proc. Natl. Acad. Sci. U.S.A..

[11] Korshunova Y.O., Eide D., Clark W.G., Guerinot M.L., Pakrasi H.B (1999). The IRT1 protein from *Arabidopsis thaliana* is a metal transporter with a broad substrate range. Plant Mol. Biol..

[12] Rogers E.E., Eide D.J., Guerinot M.L (2000). Altered selectivity in an *Arabidopsis* metal transporter. Proc. Natl. Acad. Sci. U.S.A..

[13] Abuzeineh A., Vert G., Zelazny E (2022). Birth, life and death of the *Arabidopsis* IRT1 iron transporter: the role of close friends and foes. Planta.

[14] Vert G., Briat J.F., Curie C (2001). *Arabidopsis* IRT2 gene encodes a root-periphery iron transporter. Plant J..

[15] Barberon M., Zelazny E., Robert S., Conéjéro G., Curie C., Friml J. (2011). Monoubiquitin-dependent endocytosis of the iron-regulated transporter 1 (IRT1) transporter controls iron uptake in plants. Proc. Natl. Acad. Sci. U.S.A..

[16] Spielmann J., Cointry V., Devime F., Ravanel S., Neveu J., Vert G (2022). Differential metal sensing and metal-dependent degradation of the broad spectrum root metal transporter IRT1. Plant J..

[17] Colangelo E.P., Guerinot M.L (2004). The essential basic helix-loop-helix protein FIT1 is required for the iron deficiency response. Plant Cell.

[18] Yuan Y., Wu H., Wang N., Li J., Zhao W., Du J. (2008). FIT interacts with AtbHLH38 and AtbHLH39 in regulating iron uptake gene expression for iron homeostasis in *Arabidopsis*. Cell Res..

[19] Sivitz A., Grinvalds C., Barberon M., Curie C., Vert G (2011). Proteasome-mediated turnover of the transcriptional activator FIT is required for plant iron-deficiency responses. Plant J..

[20] Selote D.S., Samira R., Matthiadis A., Gillikin J.W., Long T.A (2015). Iron-binding E3 ligase mediates iron response in plants by targeting basic helix-loop-helix transcription factors. Plant Physiol..

[21] Zhang J., Liu B., Li M., Feng D., Jin H., Wang P. (2015). The bHLH transcription factor bHLH104 interacts with IAA-LEUCINE RESISTANT3 and modulates iron homeostasis in *Arabidopsis*. Plant Cell.

[22] Li X., Zhang H., Ai Q., Liang G., Yu D (2016). Two bHLH transcription factors, bHLH34 and bHLH104, regulate iron homeostasis in *Arabidopsis thaliana*. Plant Physiol..

[23] Liang G., Zhang H., Li X., Ai Q., Yu D (2017). BHLH transcription factor bHLH115 regulates iron homeostasis in *Arabidopsis thaliana*. J. Exp. Bot..

[24] Dubeaux G., Neveu J., Zelazny E., Vert G (2018). Metal sensing by the IRT1 transporter-receptor orchestrates its own degradation and plant metal nutrition. Mol. Cell.

[25] Cointry V., Vert G (2019). The bifunctional transporter-receptor IRT1 at the heart of metal sensing and signalling. New Phytol..

[26] Bafaro E.M., Antala S., Nguyen T. V., Dzul S.P., Doyon B., Stemmler T.L. (2015). The large intracellular loop of hZIP4 is an intrinsically disordered zinc binding domain. Metallomics.

[27] Bafaro E.M., Maciejewski M.W., Hoch J.C., Dempski R.E (2019). Concomitant disorder and high-affinity zinc binding in the human zinc- and iron-regulated transport protein 4 intracellular loop. Protein Sci..

[28] Greenfield N.J., Fasman G.D (1969). Computed circular dichroism spectra for the evaluation of protein conformation. Biochemistry.

[29] Barberon M., Dubeaux G., Kolb C., Isono E., Zelazny E., Vert G (2014). Polarization of iron-regulated transporter 1 (IRT1) to the plant-soil interface plays crucial role in metal homeostasis. Proc. Natl. Acad. Sci. U.S.A..

[30] Connolly E.L., Fett J.P., Guerinot M.L (2002). Expression of the IRT1 metal transporter is controlled by metals at the levels of transcript and protein accumulation. Plant Cell.

[31] Tanaka N., Kawachi M., Fujiwara T., Maeshima M (2013). Zinc-binding and structural properties of the histidine-rich loop of *Arabidopsis thaliana* vacuolar membrane zinc transporter MTP1. FEBS Open Bio.

[32] Oldfield C.J., Dunker A.K (2014). Intrinsically disordered proteins and intrinsically disordered protein regions. Annu. Rev. Biochem..

[33] Wright P.E., Dyson H.J (2015). Intrinsically disordered proteins in cellular signalling and regulation. Nat. Rev. Mol. Cell Biol..

[34] Lanquar V., Grossmann G., Vinkenborg J.L., Merkx M., Thomine S., Frommer W.B (2014). Dynamic imaging of cytosolic zinc in *Arabidopsis* roots combining FRET sensors and RootChip technology. New Phytol..

[35] Barber-Zucker S., Shaanan B., Zarivach R (2017). Transition metal binding selectivity in proteins and its correlation with the phylogenomic classification of the cation diffusion facilitator protein family. Sci. Rep..

[36] Abramson J., Adler J., Dunger J., Evans R., Green T., Pritzel A. (2024). Accurate structure prediction of biomolecular interactions with AlphaFold 3. Nature.

[37] Lu M., Chai J., Fu D (2009). Structural basis for autoregulation of the zinc transporter YiiP. Nat. Struct. Mol. Biol..

[38] Kumar A., Ernst R.R., Wüthrich K (1980). A two-dimensional nuclear Overhauser enhancement (2D NOE) experiment for the elucidation of complete proton-proton cross-relaxation networks in biological macromolecules. Biochem. Biophys. Res. Commun..

[39] Piotto M., Saudek V., Sklenár V (1992). Gradient-tailored excitation for single-quantum NMR spectroscopy of aqueous solutions. J. Biomol. NMR.

[40] Vranken W.F., Boucher W., Stevens T.J., Fogh R.H., Pajon A., Llinas M. (2005). The CCPN data model for NMR spectroscopy: development of a software pipeline. Proteins.

[41] Güntert P., Mumenthaler C., Wüthrich K (1997). Torsion angle dynamics for NMR structure calculation with the new program DYANA. J. Mol. Biol..

[42] Schrodinger L (2010).

[43] Edelheit O., Hanukoglu A., Hanukoglu I (2009). Simple and efficient site-directed mutagenesis using two single-primer reactions in parallel to generate mutants for protein structure-function studies. BMC Biotechnol..

[44] Neveu J., Spielmann J., Fanara S., Delesalle C., Cantaloube S., Vert G (2025). Broad application of plant protein tagging with ALFA tag for nanobody-based imaging and biochemical approaches. BioRxiv.

[45] Marquès-Bueno M.D.M., Morao A.K., Cayrel A., Platre M.P., Barberon M., Caillieux E. (2016). A versatile multisite gateway-compatible promoter and transgenic line collection for cell type-specific functional genomics in *Arabidopsis*. Plant J..

[46] Karimi M., Bleys A., Vanderhaeghen R., Hilson P (2007). Building blocks for plant gene assembly. Plant Physiol..

[47] Wang Z.P., Xing H.L., Dong L., Zhang H.Y., Han C.Y., Wang X.C. (2015). Egg cell-specific promoter-controlled CRISPR/Cas9 efficiently generates homozygous mutants for multiple target genes in *Arabidopsis* in a single generation. Genome Biol..

[48] Xing H.L., Dong L., Wang Z.P., Zhang H.Y., Han C.Y., Liu B. (2014). A CRISPR/Cas9 toolkit for multiplex genome editing in plants. BMC Plant Biol..

[49] Clough S.J., Bent A.F (1998). Floral dip: a simplified method for agrobacterium-mediated transformation of *Arabidopsis thaliana*. Plant J..

[50] Murashige T., Skoog F (1962). A revised medium for rapid growth and bio assays with tobacco tissue cultures. Physiol. Plant..

[51] Jumper J., Evans R., Pritzel A., Green T., Figurnov M., Ronneberger O. (2021). Highly accurate protein structure prediction with AlphaFold. Nature.

[52] Xue B., Dunbrack R.L., Williams R.W., Dunker A.K., Uversky V.N (2010). PONDR-FIT: a meta-predictor of intrinsically disordered amino acids. Biochim. Biophys. Acta.

[53] Gupta R., Brunak S (2002). Prediction of glycosylation across the human proteome and the correlation to protein function. Pac. Symp. Biocomput..

