# Supplementary material for: Metal-sensing properties of the disordered loop from the Arabidopsis metal transceptor IRT1
Source: Biochem J. 2025 May 6;482(9):451–66. doi: 10.1042/BCJ20240685 (PMC12203963; doi:10.1042/BCJ20240685)
Supplement: online supplementary material 1 [file BCJ-482-09-BCJ20240685-s001.docx]

**Metal sensing properties of the disordered loop from the Arabidopsis metal transceptor IRT1**

**Supplemental figure legends**

**Figure S1. Predicted structure and disorder regions of the IRT1 protein.**

A) Tridimensional structure of IRT1 predicted by Alphafold. The sequence of the IRT1 loop (144-185) is shown. Disorder region is labeled in red.

B) Prediction of disordered regions obtained by PONDR®. Scores higher than 0.5 correspond to disordered regions and two arrows highlight the IRT1 loop.

**Figure S2. Residues AVGI make a turn in the IRT1 regulatory loop.**

A) Fingerprint region of the 1H-1H NOESY spectrum, showing the intramolecular and intermolecular NH-CHβ signals, recorded on the IRT1 loop. The labels in black indicate the HN(i), Hα(i) correlations and in red those corresponding to the HN(i), Hα(i-2), and HN(i), Hα(i-3) of amino acid I159.

B) Superimposition of the IRT1 loop structures calculated by the DYANA program.

C) Structure of the turn involving the three hydrophobic residues A156, V157 and I159 in licorice representation.

**Figure S3. Zinc binds to histidine residues in the IRT1 regulatory loop.**

Fingerprint region of the ^1^H-1H NOESY spectrum, recorded on the indicated two histidines double mutants of the IRT1 loop in the presence or absence of Zn^2+^, showing several intramolecular and intermolecular NH-CHβ signals (A and B) and intramolecular CHα-CHβ signals (C and D).

The sequences of the domain into which mutations have been introduced are shown above each spectrum and the mutations are highlighted in green. The spectra recorded in the absence of Zn^2+^ are shown in black and those recorded in the presence of two molar equivalents of Zn^2+^ are shown in red. Asterisks (*) indicate impurities in the sample. Annotations with a dash indicate cross-peak between inter residue i.e. NHi-CHβi-1, and without dash cross-peaks between intra residue i.e. HNi-Hβi and Hαi-Hβi.

**Figure S4. Chemical shift variations in the presence and absence of zinc.**

Chemical shift variations were determined for the wild-type (A) and indicated mutant peptides (B-E) in the absence or presence of two equivalents of Zn^2+^. Sequences of the peptides are indicated at the top of each graph. Superimposition of chemical shift variations of the mutant peptides (red) and wild-type IRT1 peptide (black) is shown.

**Figure S5. Complementation of yeast and Arabidopsis mutants with IRT1 variants.**

A) *fet3fet4* yeast transformed with the pDR195 empty vector, pDR195-IRT1 or -IRT1_D173N_, were spotted on selective media without iron or on selective media with 100 μM of Fe-EDTA. Images were taken after 4 days.

B) *fet3fet4* yeast transformed with the pDR195 empty vector, pDR195-IRT1, -IRT1_D173Q_ -IRT1_H162A/H164A_ or -IRT1_H162A/H164A/D173Q_ were spotted on selective media without iron or on selective media with 100 μM of Fe-EDTA. Images were taken after 3 days.

C) Phenotypes of Arabidopsis *irt1_crispr_*, *irt1_crispr_*/pIRT1::IRT1_H162A/H164A_-mCitrine and *irt1_crispr_*/pIRT1::IRT1_H162A/H164A/D173Q_-mCitrine. *Irt1_crispr_* mutant showed the typical chlorosis associated with loss of IRT1 while complementation with IRT1_H162A/H164A_ and with IRT1_H162A/H164A/D173Q_ restored IRT1 loss phenotype.

**Figure S6: Impact of D173N and D173Q mutations on IRT1 localization.**

A) Confocal microscopy images of epidermal cells from *Nicotiana benthamiana* plants transiently expressing IRT1 WT or mutated versions fused to mCitrine fluorescence tag under 35S promoter: 35S::IRT1_D173N_-mCitrine, 35S::IRT1_D173Q_-mCitrine and 35S::IRT1-mCitrine. IRT1 protein harboring asparagine (N) residue in the 173 position retains IRT1 at the endoplasmic reticulum while glutamine (Q) mutation allows proper IRT1 localization at the plasma membrane. Scale bars, 20 μm.

B) N-glycosylation sites predicted for IRT1_D173N_, IRT1_D173Q_ and IRT1 by NetNGlyc-1.0 server. The N residue at position 173 of IRT1 is predicted to be highly glycosylated, which could explain IRT1 retention in the endoplasmic reticulum, unlike the Q residue.

**Figure S7. In *Nicotiana benthamiana*, IRT1 endocytosis in response to non-iron metal excess relies on ubiquitination at K154 and K179 residues.**

A) Representative confocal microscopy images of epidermal cells from *Nicotiana benthamiana* leaves transiently expressing 35S::IRT1_K154R/K179R_-mCitrine after 3 hours of control treatment (without non-iron metals; - metals) or after non-iron metal excess treatment (+++ metals). Showed maximum projection of 10-15 optical sections taken using 1 µm z-distance. Scale bars, 20 µm.

(B) Quantification of the ratio of the plasma membrane to intracellular signals and (C) quantification of intracellular particles per µm^2^ from cells exposed to metal excess relative to cells exposed to control solution of plants treated as described in A). Error bars represent SD (n=40-50) from 3 independent experiments. ‘ns’ indicate no significant differences (one-way ANOVA, Tukey post-test).

**Figure S8: Zinc coordination by the triple mutated IRT1 regulatory loop.**

Alphafold3 prediction for the IRT1 loop triple mutant H162A/H164A/D173N coordinating Zn^2+^ ion (grey sphere) with residues H166, H168 and D144. Slashed green lines represent metal coordination within 3.5 Å distance. Analyses performed with USCF ChimeraX

**Figure S9: Microscale thermophoresis data for Zn^2+^ binding.**

Microscale thermophoresis analyses of zinc binding by the wild-type IRT1 loop (IRT1; dark blue), single mutant with aspartic acid 173 mutated to asparagine (D173N_;_ yellow), double mutant with histidine residues 162 and 164 mutated to alanine (H162A/H164A_;_ green), double mutant with histidine residues 166 and 168 mutated to alanine (H166A/H168A; orange), triple mutant with histidine residues 162 and 164 mutated to alanine and aspartic acid 173 mutated to asparagine (H162A/H164A/D173N; red), and quadruple mutant with histidine residues 162, 164, 166 and 168 mutated to alanine (4HA; light blue). Dots represent the average dose response of at least six technical replicates derived from two biological replicates. Table shows MST binding parameters comprising Standard Error of Regression, Signal to Noise and Response Amplitude values.

**Figure S10: Microscale thermophoresis data for Mn^2+^ binding.**

Microscale thermophoresis analyses of manganese binding by the wild-type IRT1 loop (IRT1; dark pink), single mutant with aspartic acid 173 mutated to asparagine (D173N_;_ yellow), double mutant with histidine residues 162 and 164 mutated to alanine (H162A/H164A_;_ green) double mutant with histidine residues 166 and 168 mutated to alanine (H166A/H168A; orange), triple mutant with histidine residues 162 and 164 mutated to alanine and aspartic acid 173 mutated to asparagine (H162A/H164A/D173N; red), and quadruple mutant with histidine residues 162, 164, 166 and 168 mutated to alanine (4HA; light pink). Dots represent the average dose response of at least six technical replicates derived from two biological replicates. Table shows MST binding parameters comprising Standard Error of Regression, Signal to Noise and Response Amplitude values.
