# Supplementary figures and images for: Metal-sensing properties of the disordered loop from the Arabidopsis metal transceptor IRT1
Source: Biochem J. 2025 May 6;482(9):451–66. doi: 10.1042/BCJ20240685 (PMC12203963; doi:10.1042/BCJ20240685)

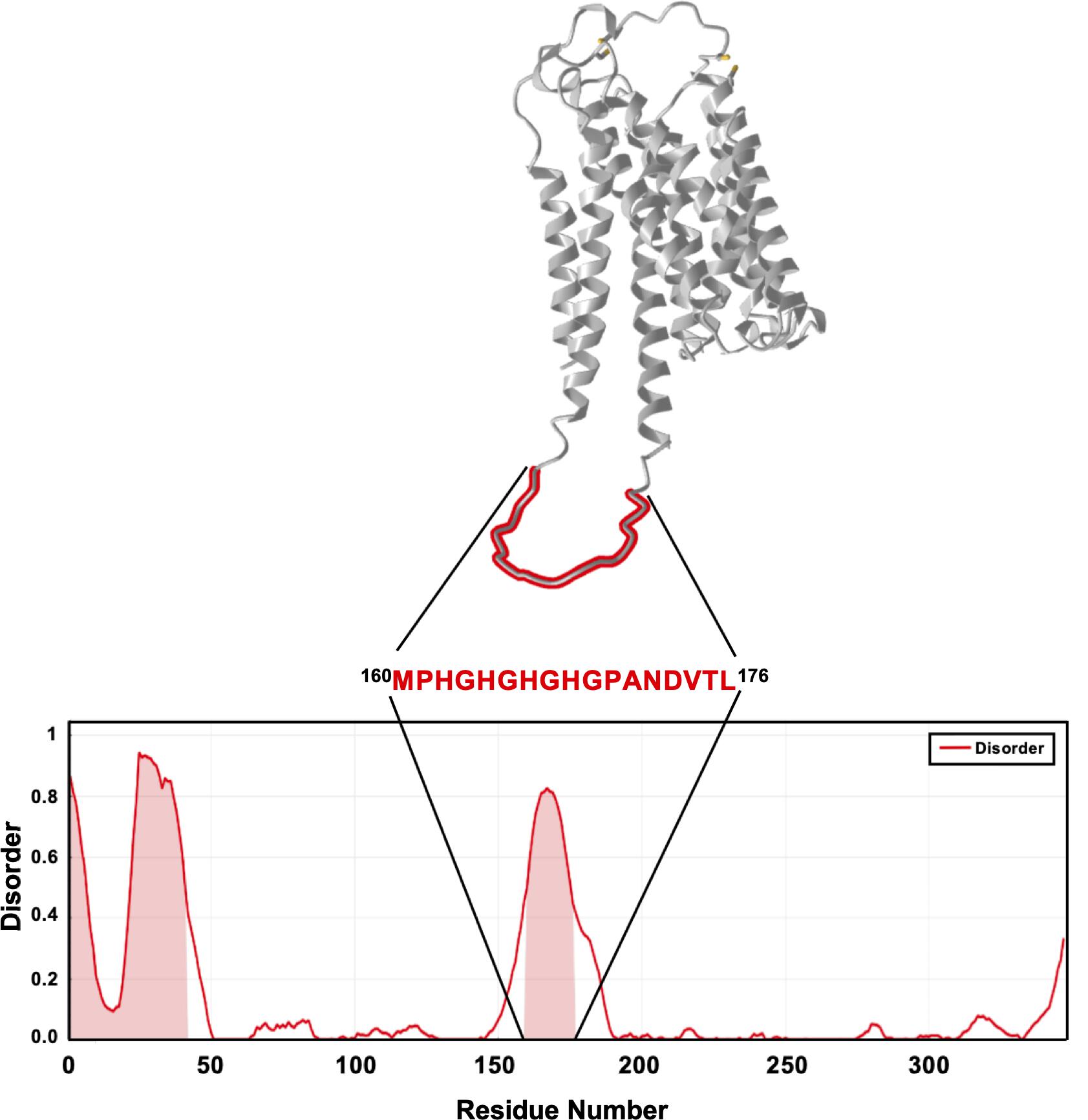

Supplement: Online supplementary figure 1 [file BCJ-482-09-BCJ20240685-s001.jpg]

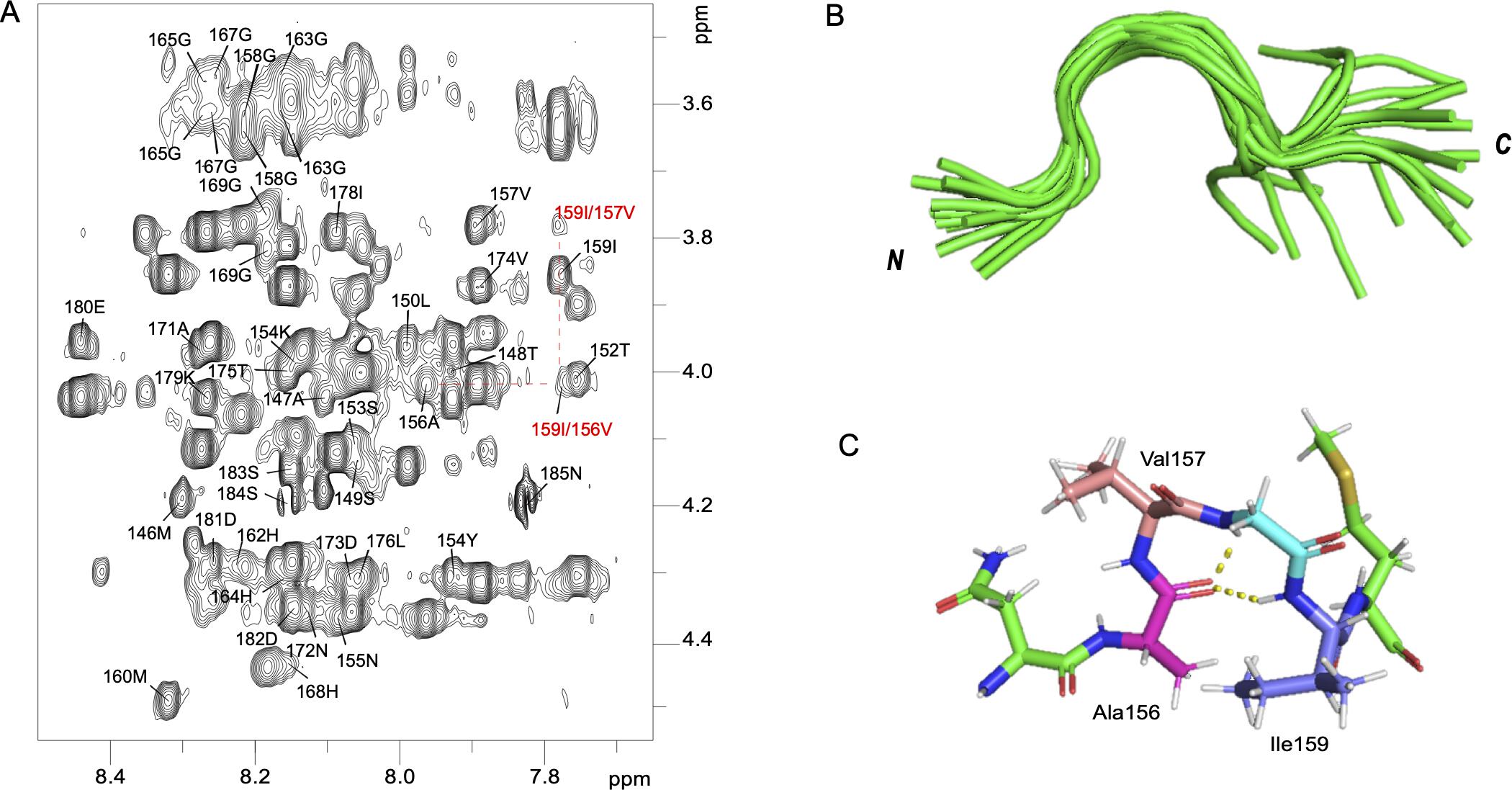

Supplement: Online supplementary figure 2 [file BCJ-482-09-BCJ20240685-s002.jpg]

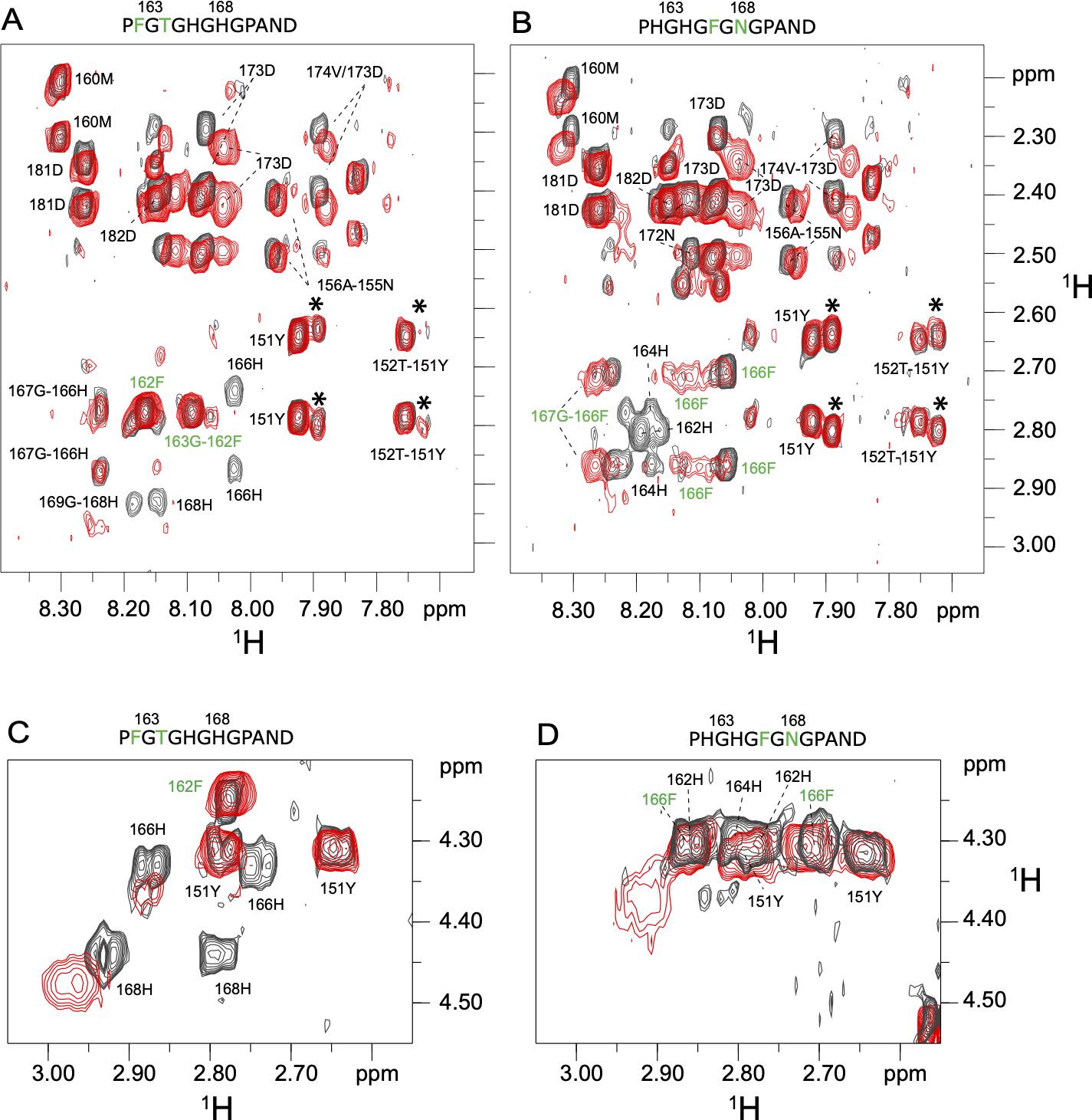

Supplement: Online supplementary figure 3 [file BCJ-482-09-BCJ20240685-s003.jpg]

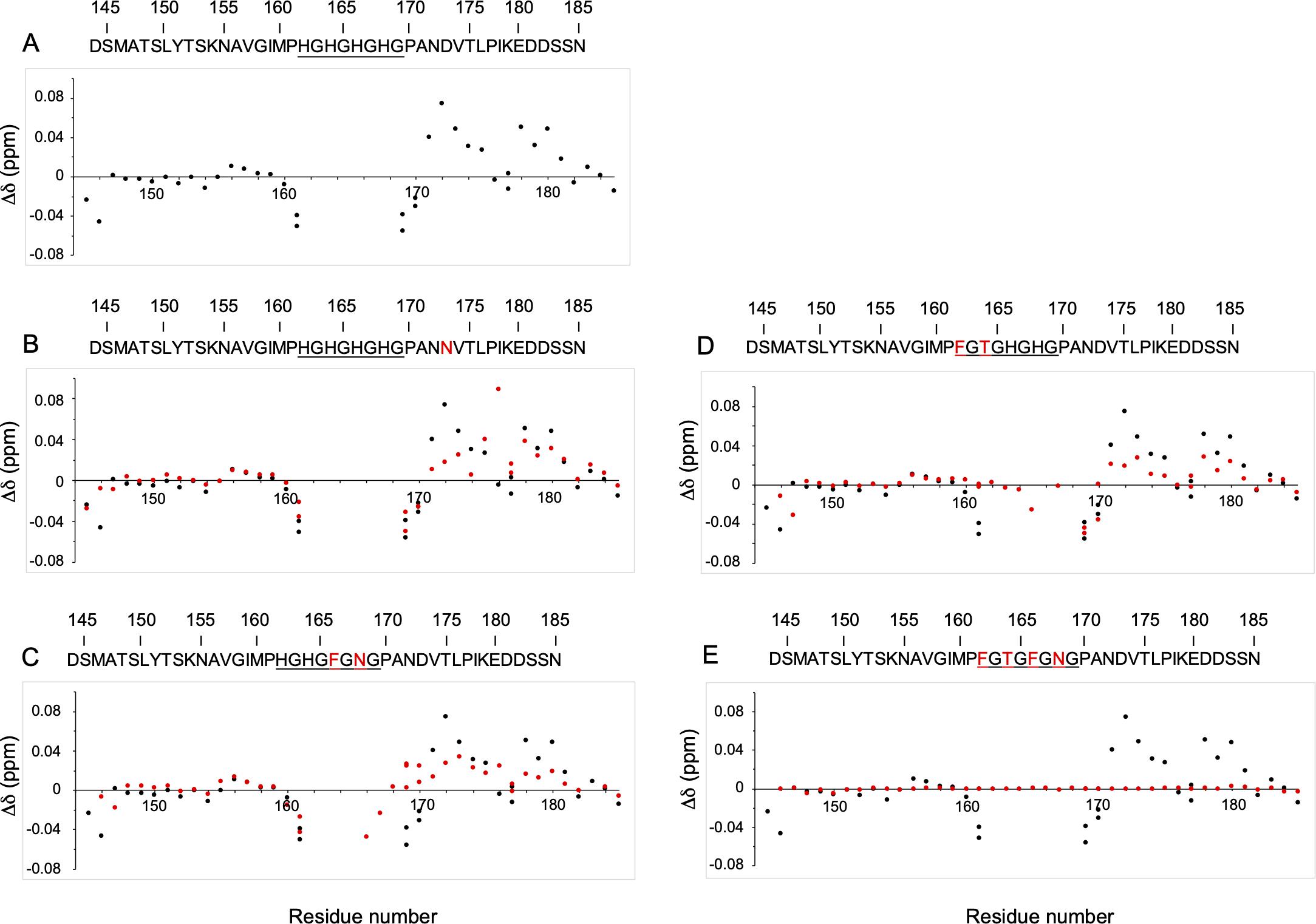

Supplement: Online supplementary figure 4 [file BCJ-482-09-BCJ20240685-s004.jpg]

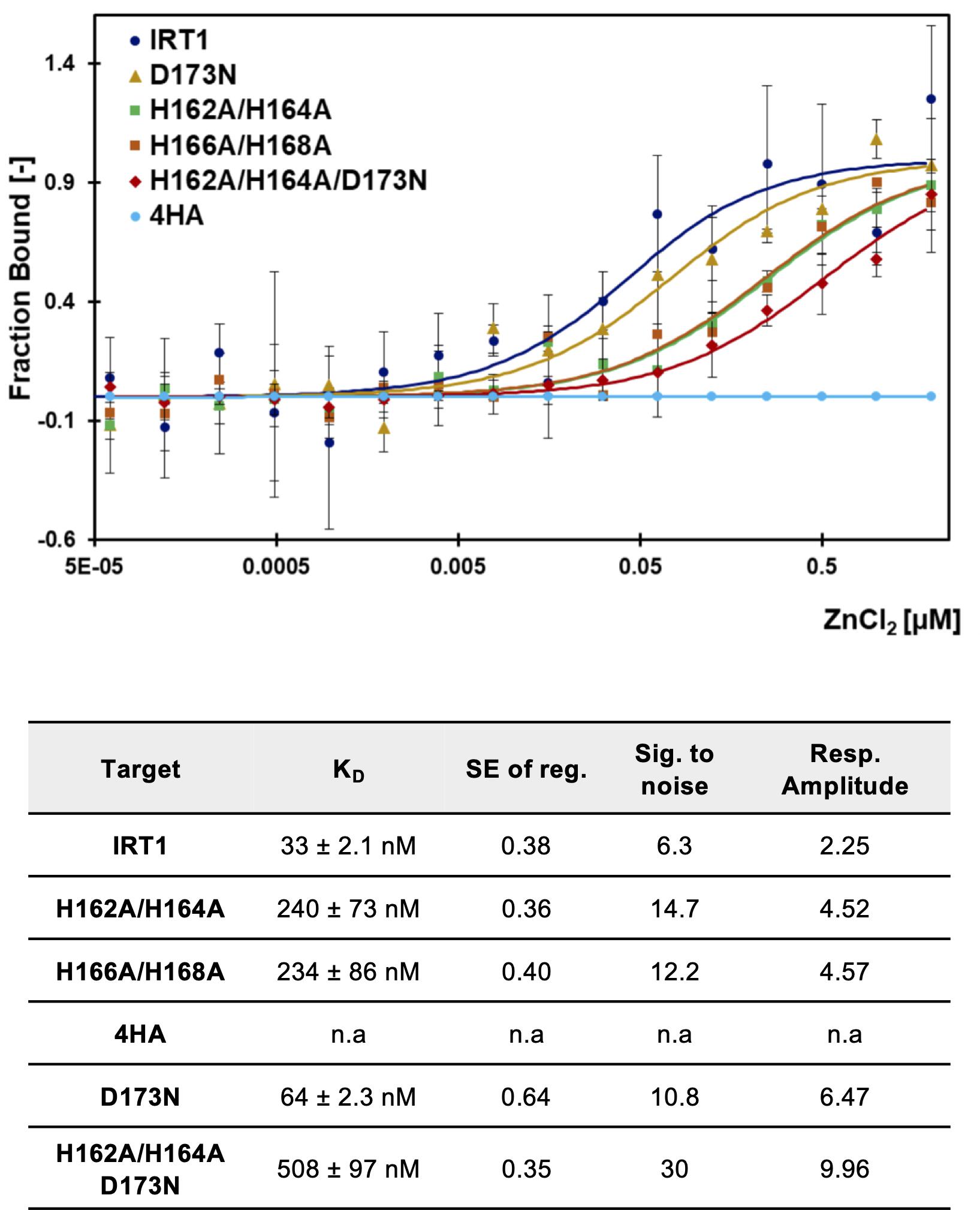

Supplement: Online supplementary figure 5 [file BCJ-482-09-BCJ20240685-s005.jpg]

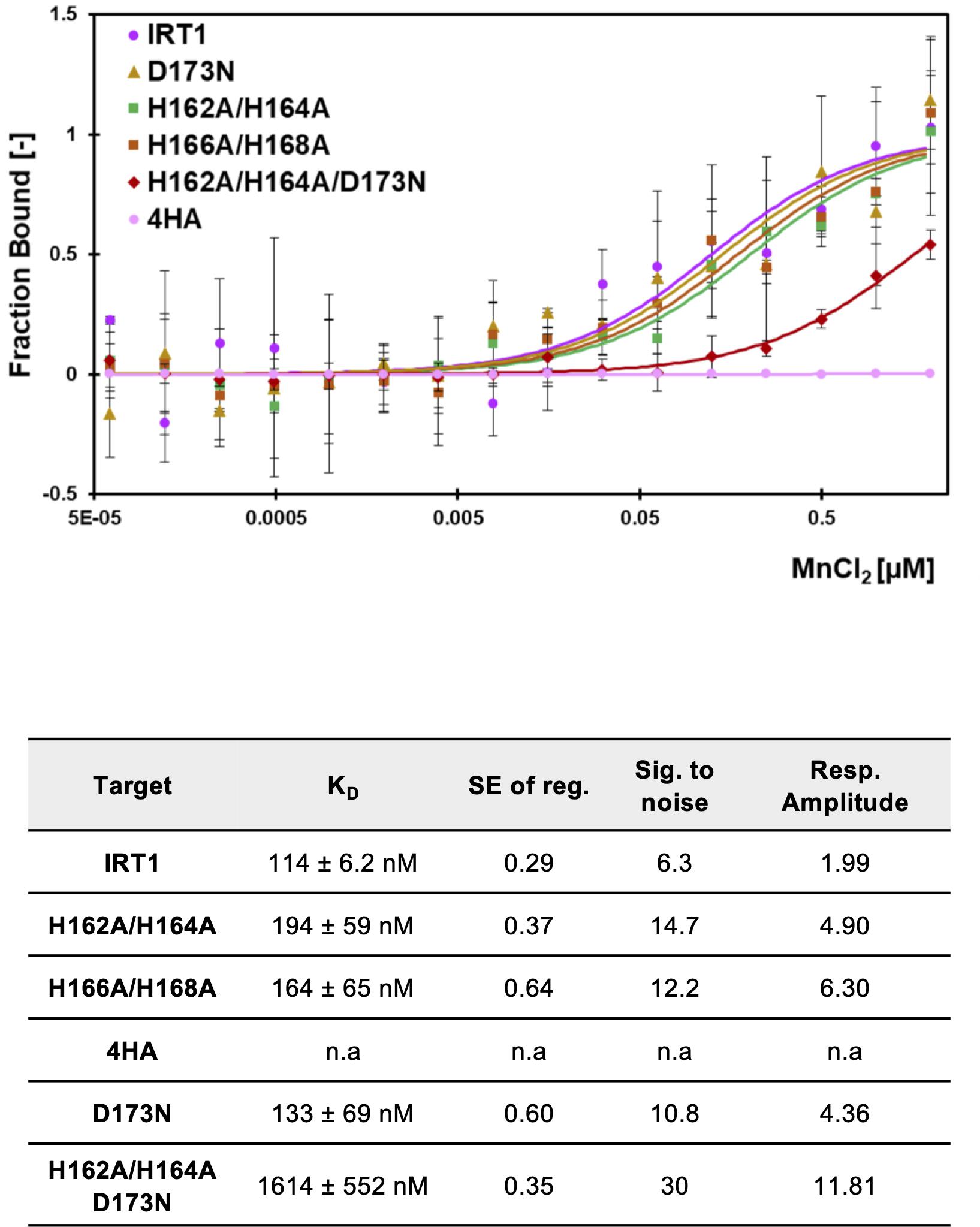

Supplement: Online supplementary figure 6 [file BCJ-482-09-BCJ20240685-s006.jpg]

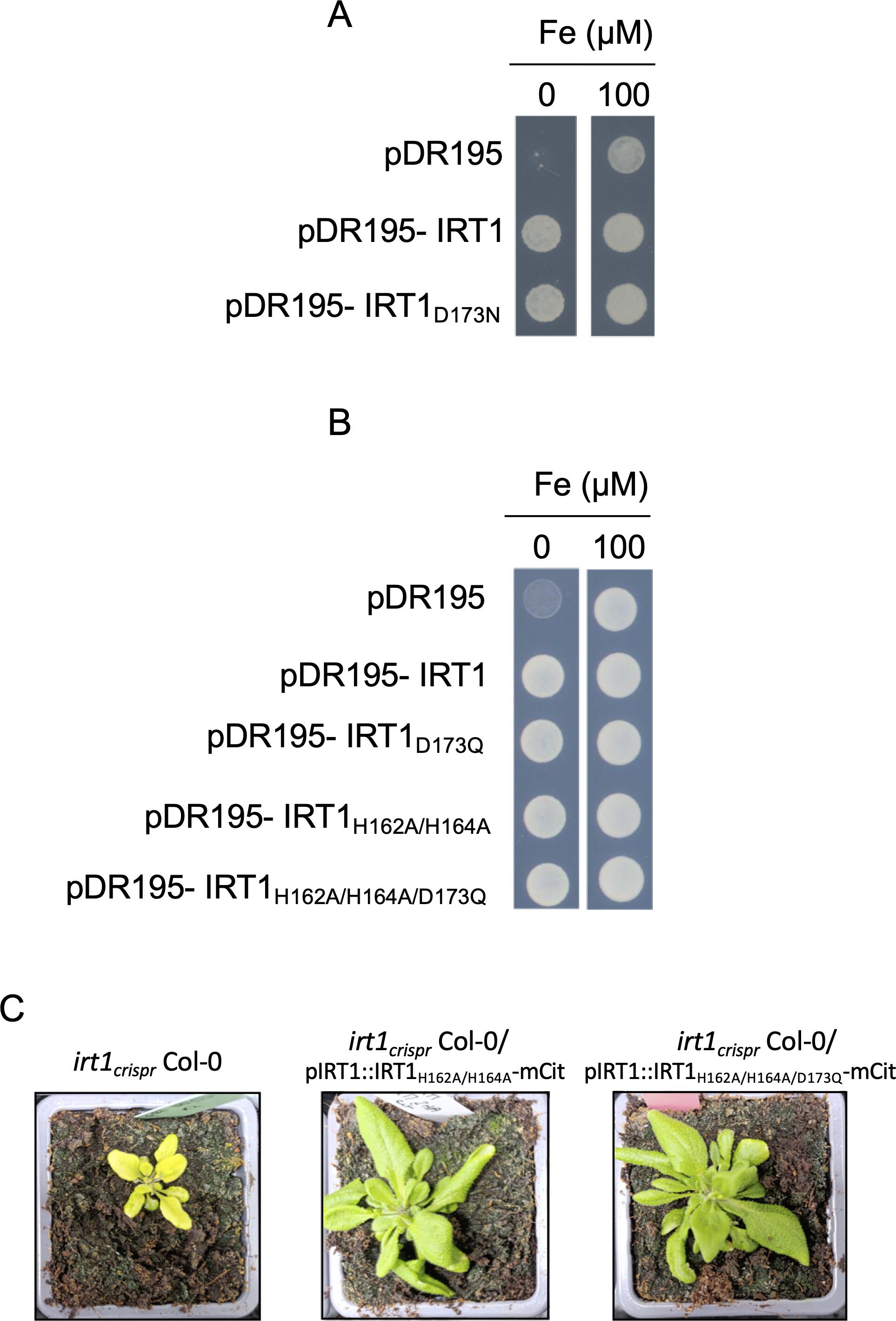

Supplement: Online supplementary figure 7 [file BCJ-482-09-BCJ20240685-s007.jpg]

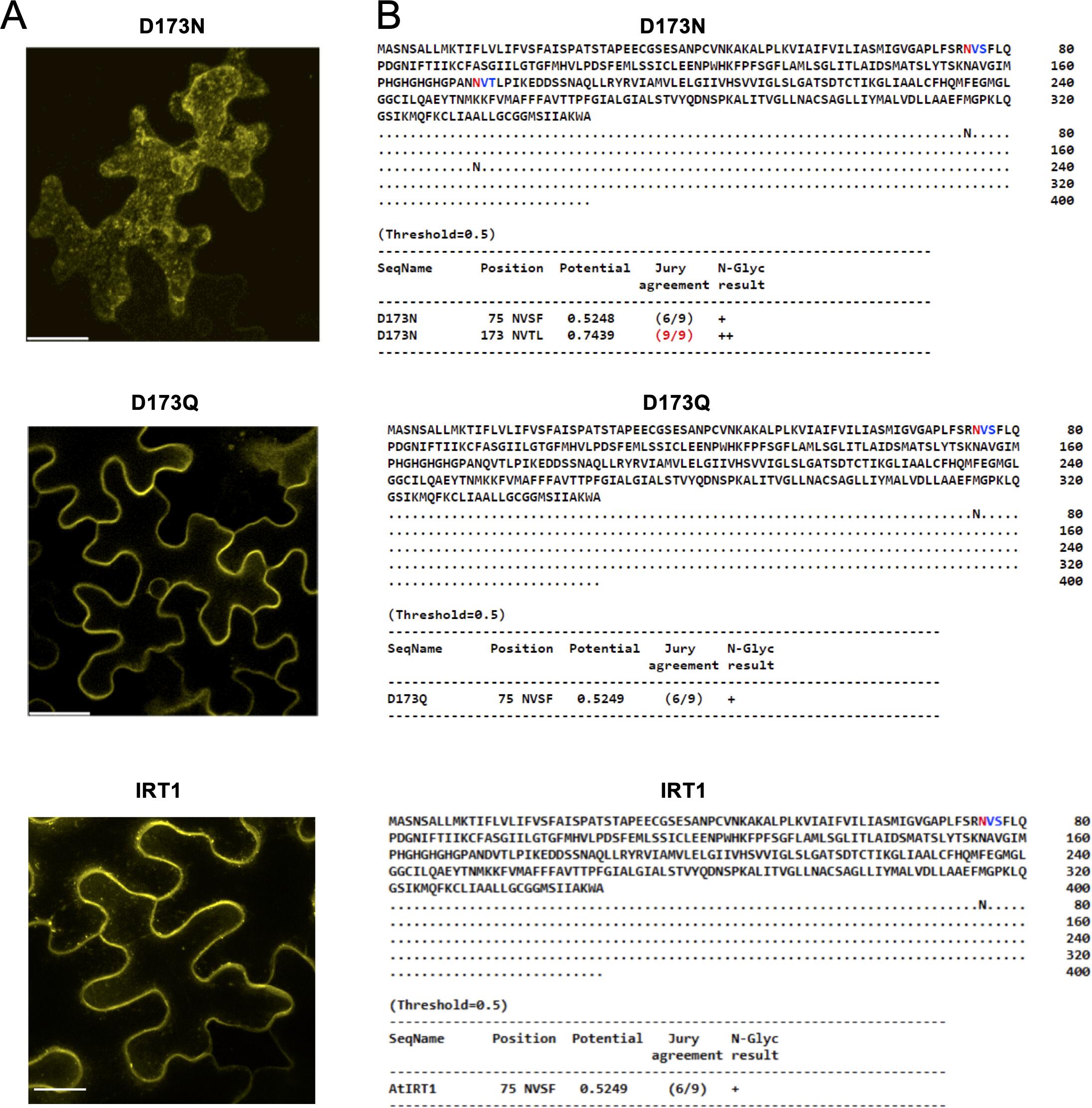

Supplement: Online supplementary figure 8 [file BCJ-482-09-BCJ20240685-s008.jpg]

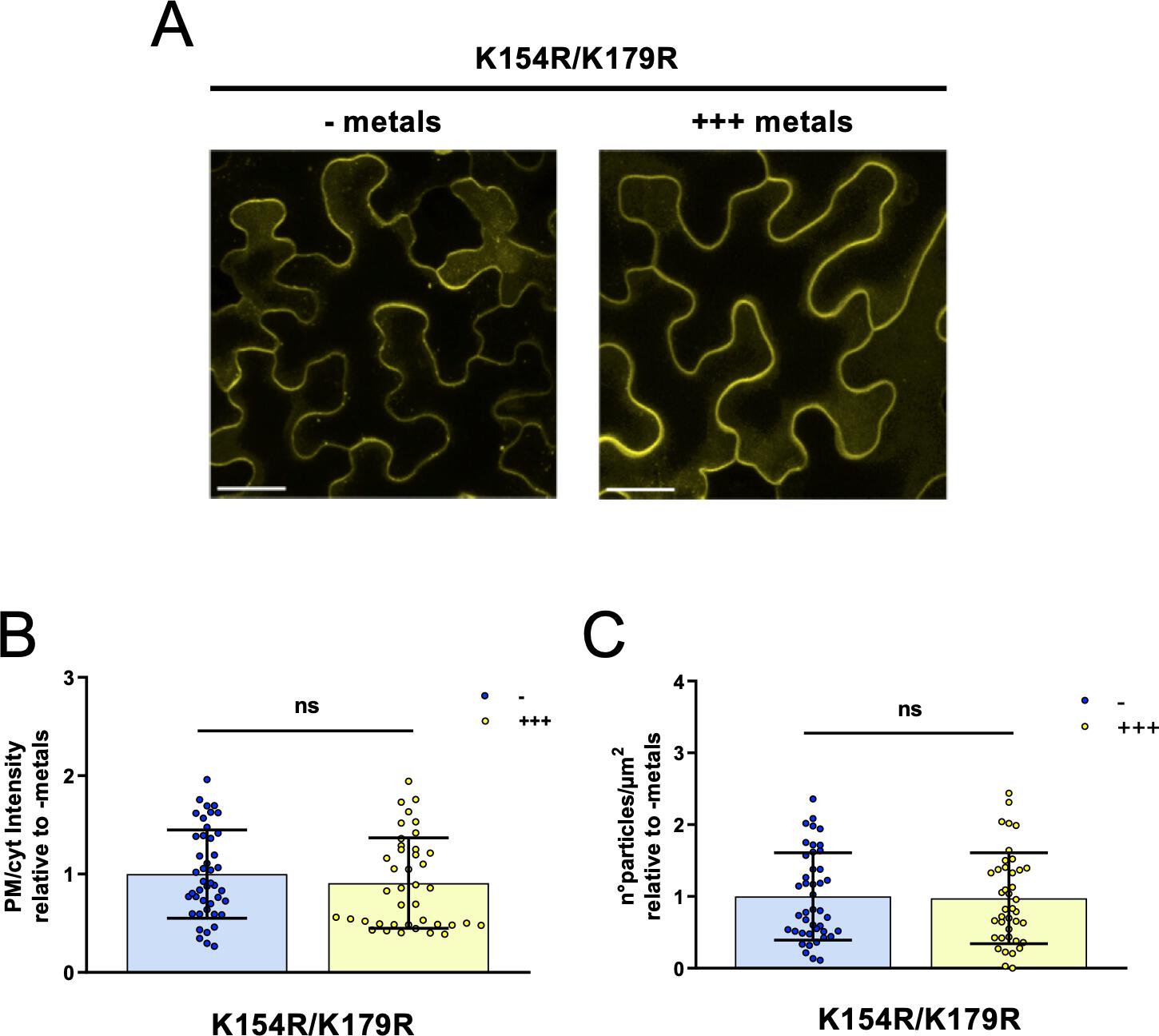

Supplement: Online supplementary figure 9 [file BCJ-482-09-BCJ20240685-s009.jpg]

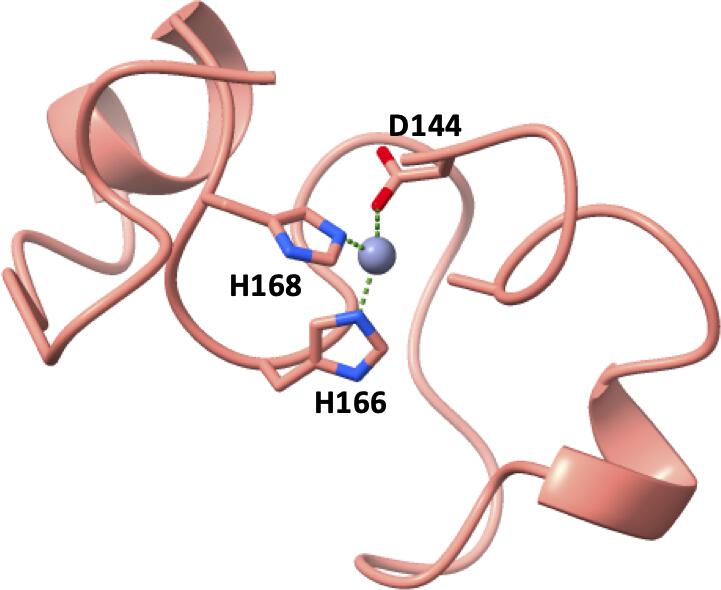

Supplement: Online supplementary figure 10 [file BCJ-482-09-BCJ20240685-s010.jpg]

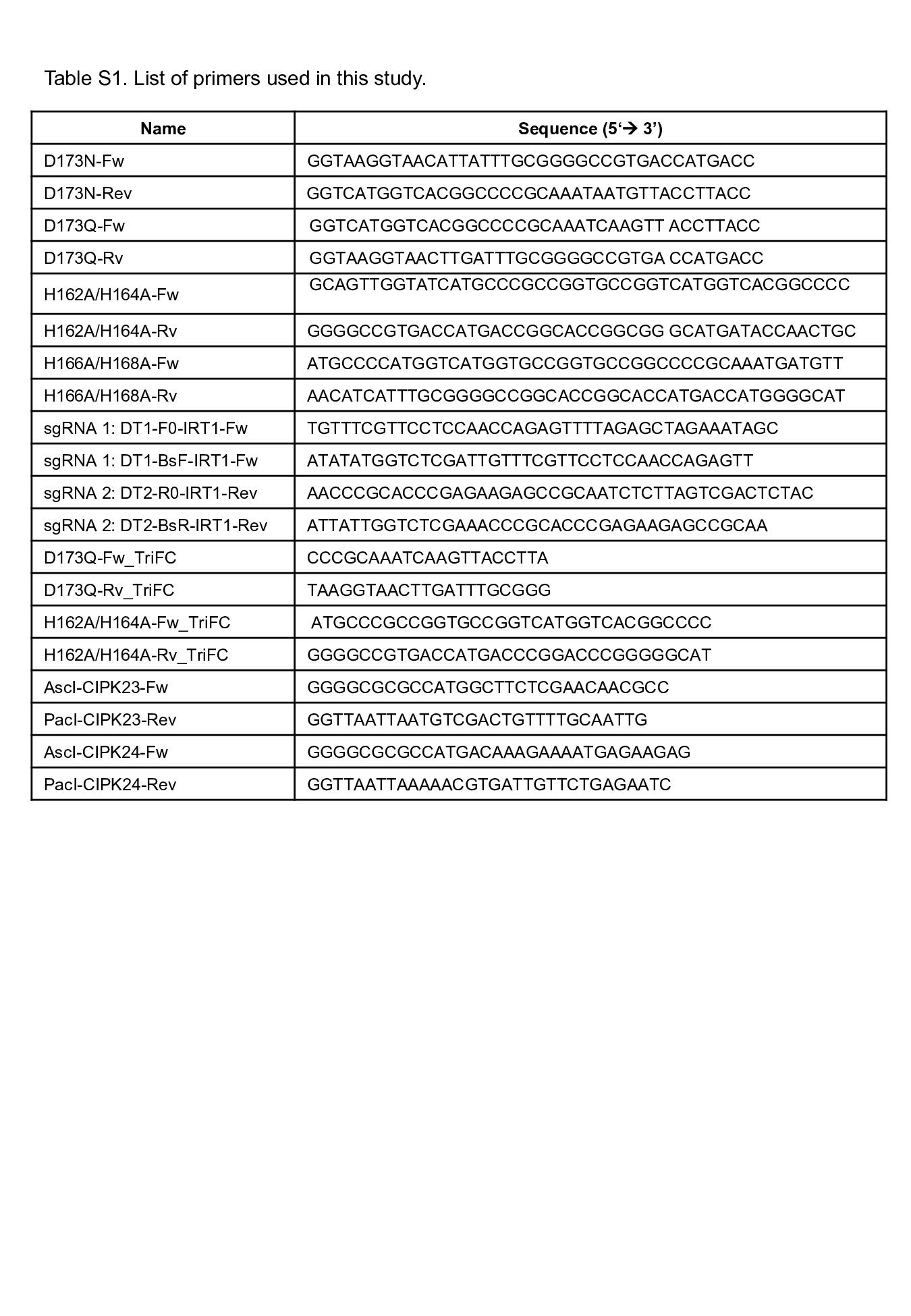

Supplement: Online supplementary table 1 [file BCJ-482-09-BCJ20240685-s012.jpg]

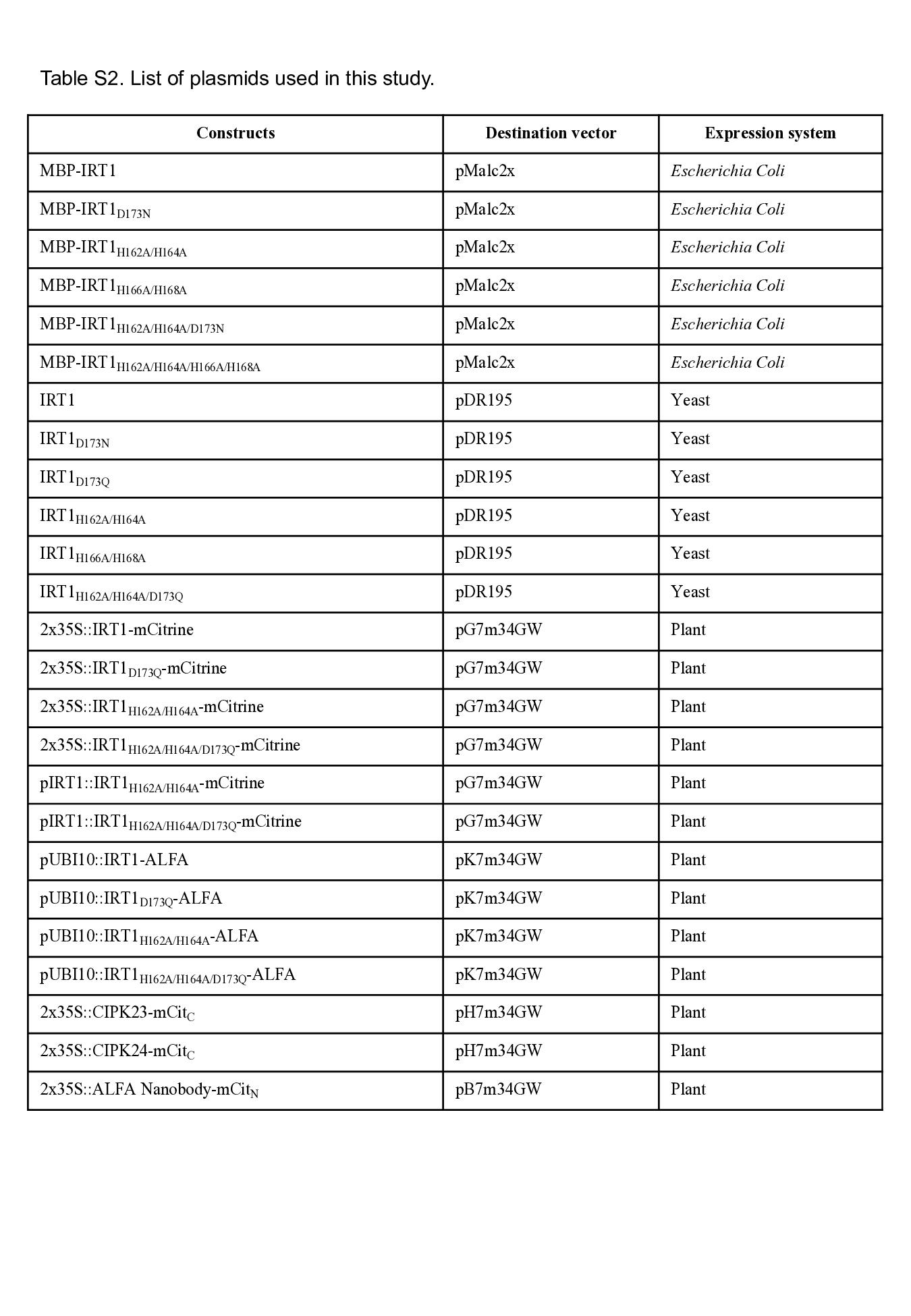

Supplement: Online supplementary table 2 [file BCJ-482-09-BCJ20240685-s013.jpg]
